# Development of a multi-epitope vaccine candidate for leishmanial parasites applying immunoinformatics and *in vitro* approaches

**DOI:** 10.3389/fimmu.2023.1269774

**Published:** 2023-11-15

**Authors:** Jyotisha, Rahila Qureshi, Insaf Ahmed Qureshi

**Affiliations:** ^1^ Department of Biotechnology & Bioinformatics, School of Life Sciences, University of Hyderabad, Hyderabad, India; ^2^ Centre for DNA Fingerprinting and Diagnostics, Hyderabad, India

**Keywords:** leishmaniasis, membrane-bound acid phosphatase, vaccine development, mammalian macrophage, immune response

## Abstract

Leishmaniasis is a neglected tropical disease, and its severity necessitates the development of a potent and efficient vaccine for the disease; however, no human vaccine has yet been approved for clinical use. This study aims to design and evaluate a multi-epitope vaccine against the leishmanial parasite by utilizing helper T-lymphocyte (HTL), cytotoxic T-lymphocyte (CTL), and linear B-lymphocyte (LBL) epitopes from membrane-bound acid phosphatase of *Leishmania donovani* (*Ld*MAcP). The designed multi-epitope vaccine (*Ld*MAPV) was highly antigenic, non-allergenic, and non-toxic, with suitable physicochemical properties. The three-dimensional structure of *Ld*MAPV was modeled and validated, succeeded by molecular docking and molecular dynamics simulation (MDS) studies that confirmed the high binding affinity and stable interactions between human toll-like receptors and *Ld*MAPV. *In silico* disulfide engineering provided improved stability to *Ld*MAPV, whereas immune simulation displayed the induction of both immune responses, i.e., antibody and cell-mediated immune responses, with a rise in cytokines. Furthermore, *Ld*MAPV sequence was codon optimized and cloned into the pET-28a vector, followed by its expression in a bacterial host. The recombinant protein was purified using affinity chromatography and subjected to determine its effect on cytotoxicity, cytokines, and nitric oxide generation by mammalian macrophages. Altogether, this report provides a multi-epitope vaccine candidate from a leishmanial protein participating in parasitic virulence that has shown its potency to be a promising vaccine candidate against leishmanial parasites.

## 1 Introduction

Leishmaniasis is a vector-borne protozoan disease caused by the leishmanial parasite that primarily affects the poorest and most vulnerable people globally. Among various disease forms, visceral leishmaniasis (VL) is the most severe form caused by *Leishmania donovani* and *L. infantum*. Every year, an estimated 50,000–90,000 new cases of VL are diagnosed worldwide, and the disease is endemic in over 60 countries (1). Currently available treatments are based on chemotherapy, but the drugs are mostly toxic, and cause serious side effects along with an increased incidence of drug resistance (2, 3). However, a vaccine-based approach to controlling disease is a viable option, as most of the people who recover from VL develop immune protection against leishmaniasis and remain resistant to subsequent clinical reinfection for a considerable time (4). In addition, vaccines are considered less expensive and safer than other treatments. Nonetheless, no licensed vaccine is currently available for clinical use to prevent leishmanial infection in humans, which advocates for the dire need for a potent vaccine to combat the disease (5).

Several approaches have been followed to design vaccines for VL using individual or recombinant antigens of parasites, cellular extracts, and live-attenuated or killed parasites. The only type of human preventive VL vaccine that has entered phase III clinical trials thus far is the first-generation vaccine; however, the results of this vaccine were unsatisfactory (6). The recombinant leishmanial antigens, i.e., single peptides or polypeptides, are employed to produce the second-generation vaccine. A multi-component vaccine (LEISH-F3) adjuvanted with GLA-SE was one of the candidates that exhibited encouraging outcomes in the phase I trial (7). A third-generation DNA vaccine (ChAd63-KH) has demonstrated its potential in a phase I clinical trial as a safe and immunogenic therapeutic vaccine against VL and post-kala azar dermal leishmaniasis (PKDL) (8). Despite the current progress in vaccine development, the precedence goal of developing a safe, effective, long-lasting, and low-cost preventive vaccine against VL has yet to be achieved (9). In comparison to conventional vaccines, epitope-based chimeric or subunit vaccines have numerous advantages as they do not contain the whole pathogen, either live or killed, and exhibit high specificity and stability (10). Therefore, the epitope mapping of immunogenic proteins is pivotal in developing peptide vaccines. Through proteome studies, it has been established that leishmanial parasites consist of many vital immunogenic candidates (11). These proteins are widely associated with infection and pathogenicity. Glycoprotein 63 (gp63) (12, 13), KMP-11 (14), A2 protein (15), and *Leishmania*-activated C kinase (LACK) (16) have been used as a peptide-based vaccine for experimental studies; however, their further validation through human trials is yet to come (17–19).

**Figure 1:**
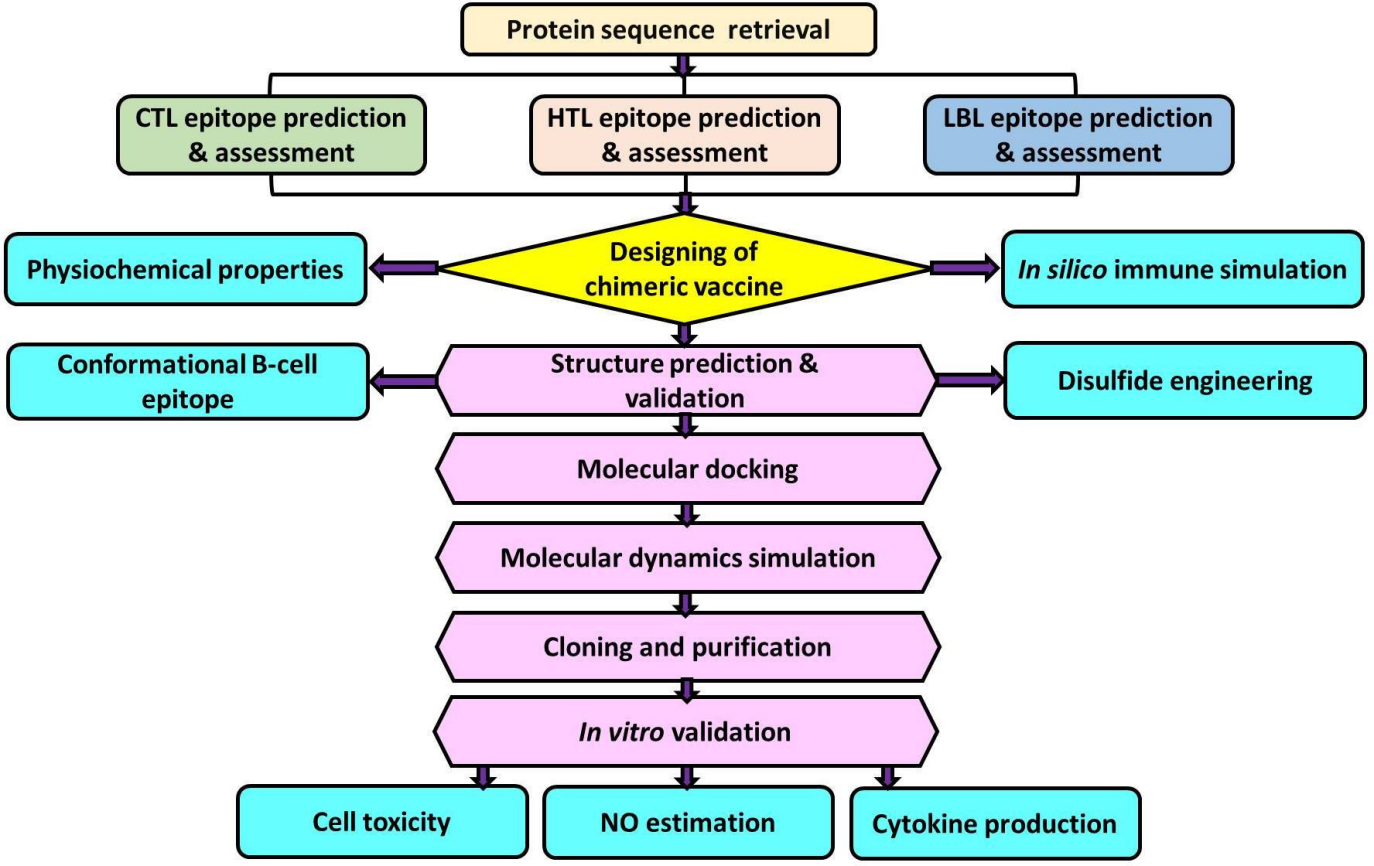
Schematic representation of multi-epitope vaccine designing from *Leishmania donovani* membrane-bound acid phosphatase (*Ld*MAcP) and its validation in mammalian macrophages.

For many years, endogenous leishmanial phosphatases have been studied in the context of infection. Although their functions are not fully understood, numerous studies indicate that they are crucial for parasitic virulence and resistance to stress environments in the life cycle of *Leishmania* (20). For instance, the histidine acid phosphatase (HAcP) of *L. amazonensis* was reported to be involved in an upsurge in the association index of parasite-macrophage (21). Several recent studies employing gene ablation and/or overexpression in different species of *Leishmania* have established the contribution of various phosphatases to the survival of the parasite (22–25). Membrane-bound acid phosphatase (*Ld*MAcP), a member of HAcP, is associated with host-pathogen interactions. It is essential for parasitic virulence as it aids in adapting to acidic conditions, acquiring resources from phosphorylated substrates in the host cell, and the parasite’s survival (22). Besides, *Ld*MAcP is expressed on the surface membranes of the promastigote form, and epitopes derived from it are readily accessible to both humoral and cell-mediated immunity (26) thus, this protein might be extremely useful for developing a noble vaccine. Therefore, the aim of our study was to design and analyze a multi-epitope vaccine candidate utilizing epitopes screened from the *Ld*MAcP protein through bioinformatic tools. The vaccine construct designed here confers substantial immunogenic ability, which has been further validated through *in vitro* studies (**Figure 1**).

## 2 Materials and methods

### 2.1 Retrieval of target protein sequence

The protein sequence of *Ld*MAcP was retrieved from UniProtKB (ID: A0A075IPV3) in FASTA format to identify potential epitopes for designing a multi-epitope based chimeric vaccine. The protein antigenicity was assessed considering the parasite as a target organism with a threshold value of 0.4 on VaxiJen v2.0 (http://www.ddg-pharmfac.net/vaxijen/VaxiJen/VaxiJen.html) web server. Additionally, its allergenicity, exomembrane topology, and various physicochemical properties were analyzed with the help of online tools such as AllerTOP v. 2.0 (https://www.ddg-pharmfac.net/AllerTOP/), TMHMM v0.2 (http://www.cbs.dtu.dk/services/TMHMM/), and ExPASy ProtParam (https://web.expasy.org/protparam/), respectively.

### 2.2 Prediction and assessment of T-cell epitopes

The sequence-based screening server, NetCTL v1.2 (http://www.cbs.dtu.dk/services/NetCTL/), was utilized to find CTL epitopes from *Ld*MAcP protein for all 12 MHC-I supertypes. All the 12 MHC-I supertypes were included in the study to cover a diverse range of MHC-I alleles that are prevalent across various populations. The threshold value of 0.75, with corresponding sensitivity and precision of 0.80 and 0.97, was used for the prediction. All other parameters were set to default to provide optimal predictive performance. Afterward, the consensus method of IEDB class I MHC binding prediction online tool (http://tools.iedb.org/mhci/) was applied to identify both types of frequent and non-frequent class I MHC binding alleles taking <2 percentile rank under consideration as lower percentile ranks indicate high binding affinity of epitopes for alleles. For these alleles, human was taken into account as the source species. Finally, the epitopes that not only met this threshold but also interacted with at least 3 different MHC-I alleles were considered for subsequent analysis. Simultaneously, the NN_Align method of IEDB web database (http://tools.iedb.org/mhcii/) was used to find out MHC-II restricted 15-mer long HTL epitopes by considering an IC50 value of <20 nm and a percentile rank of <2 that indicate a higher binding affinity for HTL epitopes.

The predicted CTL and HTL epitopes were further examined to select the best epitopes based on their essential features, such as antigenicity, non-allergenicity, and toxicity, by VaxiJen v2.0, AllerTOP v. 2.0, and ToxinPred server (http://crdd.osdd.net/raghava/toxinpred/), respectively. Additionally, the immunogenicity of screened CTL epitopes was predicted using the MHC-I immunogenicity tool of IEDB (http://tools.iedb.org/immunogenicity/). As IFN-γ stimulates macrophages and natural killer cells to provide a selective response to MHC antigens in innate and acquired immune responses, the predicted HTL epitopes were submitted to the IFNepitope web server (http://crdd.osdd.net/raghava/ifnepitope/) for determination of their ability to induce IFN-γ production. The Support Vector Machine (SVM) and motif-based hybrid approach were employed with standard parameters for segregation into IFN-γ and non-IFN-γ inducing epitopes.

### 2.3 Identification and selection of linear B-lymphocyte (LBL) epitopes

The LBL epitopes were identified from *Ld*MAcP sequence through ABCpred (http://crdd.osdd.net/raghava/abcpred/) and BepiPred (https://services.healthtech.dtu.dk/service.php?BepiPred-2.0) servers, considering 0.5 as the standard threshold value. Epitopes common to both tools were further investigated for other properties, including transmembrane topology, antigenicity, non-allergenicity, and non-toxicity. Afterward, the protein sequence was subjected to Emini surface accessibility, Chou and Fasman beta-turn, Parker hydrophilicity, and Karplus and Schulz flexibility prediction methods in IEDB (http://tools.iedb.org/bcell/) to analyze various parameters of selected epitopes. A Default threshold value with a window size of 7 amino acids was used for all four prediction methods, except Emini surface accessibility, in which 6 amino acids were considered.

### 2.4 Prediction of population coverage and cross-reactivity assessment

The population coverage tool of IEDB analysis resource (http://tools.iedb.org/population/) was implemented for the determination of the population coverage in countries with high rates of visceral leishmaniasis; hence, T-cell epitopes with respective HLA alleles were contemplated. In addition, selected CTL, HTL, and LBL epitopes were submitted to the Multiple Peptide Match tool of Protein Information Resource (https://research.bioinformatics.udel.edu/peptidematch/batchpeptidematch.jsp) to assess the similarities between selected epitopes and the human proteome.

### 2.5 Designing and characterization of vaccine construct

The multi-epitope based chimeric vaccine (*Ld*MAPV) was formulated by conjugating the selected CTL, HTL, and LBL epitopes with AAY and GDGDG linkers. Moreover, synthetic peptide RS-09 (APPHALS), a TLR4 agonist, was chosen here as an adjuvant and incorporated at the N-terminus of the vaccine candidate associated with the first CTL epitope through the EAAAK linker. To access the antigenicity and non-allergenicity of *Ld*MAPV sequence, the corresponding Vaxijen v2.0 and AllerTOP v2.0 prediction tools were used. For further confirmation of allergenic property with high accuracy, the AllergenFP v1.0 (http://ddg-pharmfac.net/AllergenFP/) server was utilized. In addition, the toxicity of the designed *Ld*MAPV was checked by the ToxinPred server. Simultaneously, an assessment of different physicochemical characteristics of the *Ld*MAPV construct was done by the ExPASy ProtParam server. Protein-Sol (https://protein-sol.manchester.ac.uk/) and SOLpro (https://scratch.proteomics.ics.uci.edu/) servers were also employed to estimate the solubility of *Ld*MAPV after overexpression in *E. coli*. Moreover, the solvent-accessible feature of *Ld*MAPV was analyzed using the RaptorX Property server (http://raptorx.uchicago.edu/StructurePropertyPred/predict/).

### 2.6 Structure prediction and validation of *Ld*MAPV

The percentage of secondary structural contents of *Ld*MAPV was computed through the PsiPred 4.0 server (http://bioinf.cs.ucl.ac.uk/psipred/) and PDBsum (http://www.ebi.ac.uk/thornton-srv/databases/cgi-bin/pdbsum/GetPage.pl?pdbcode=index.html), keeping all the parameters at default. The three-dimensional structure of *Ld*MAPV was generated employing threading and *ab initio* approaches through the Iterative Threading Assembly Refinement (I-TASSER) online server (https://zhanggroup.org/I-TASSER/). Furthermore, for improvement in the predicted 3D model, the selected *Ld*MAPV structure was submitted to a two-step model refinement proedure through GalaxyLoop and GalaxyRefine on the GalaxyWEB server (https://galaxy.seoklab.org/index.html). Successively, Procheck (https://servicesn.mbi.ucla.edu/PROCHECK/), ProSAweb (https://prosa.services.came.sbg.ac.at/prosa.php), and ERRAT (https://servicesn.mbi.ucla.edu/ERRAT/) web tools were used for quality assessment of the modeled structure.

### 2.7 Discontinuous B-cell epitope prediction

The discontinuous or conformational B-cell epitopes were obtained by the Ellipro web-based server (https://tools.iedb.org/ellipro/help/) by providing a refined and validated 3D model of *Ld*MAPV as input with a minimum score of 0.5 and a maximum distance of 6. It is a structure-based approach and uses modified Thornton’s method to predict the antibody epitopes.

### 2.8 Disulfide engineering for vaccine stability

To enhance the stability of three-dimensional structure of the vaccine, the web server Disulfide by Design v2.12 (http://cptweb.cpt.wayne.edu/DbD2/) was used to generate disulfide bonds between potential residue pairs. The residue pairs with energy <2.5 (kcal/mol) and Chi^3^ (χ^3^) between −87 and +97 degrees were considered for disulfide engineering. Further, the engineered and wild-type *Ld*MAPV were subjected to MD simulation for analysis of protein stability. All the MD simulations were executed with the CHARMM36 force field (27) of the Linux-based GROMACS 5.1.4 software (28). Protein solvation was performed by placing these systems in a cubic box and solvating them with the TIP3P water model (29). After neutralizing the system with appropriate counter ions, the steepest descent algorithm was applied to minimize the energy of the system in 50000 steps. Following the energy minimization step, each system was subjected to 1 ns of NVT (at 300 K) and NPT (at 1 bar) ensemble equilibration, and then a 100 ns production simulation was done for all the equilibrated systems. Consequently, the root mean square deviation (RMSD), root mean square fluctuation (RMSF), and radius of gyration (Rg) were calculated employing the production simulation data, as mentioned earlier (30).

### 2.9 Interaction analysis of vaccine-TLR complexes

One of the *in silico* approaches applied to estimate the binding affinity and interaction pattern between receptor and ligand in the complex is molecular docking. Hence, the crystal structures of the TLR4/MD2 complex (PDB ID: 3FXI) and TLR2 (PDB ID: 2Z7X) were obtained from the Protein Data Bank (https://www.rcsb.org/). The protein-protein docking server ClusPro 2.0 (https://cluspro.bu.edu/login.php) was used to perform molecular docking between the modeled *Ld*MAPV structure as a ligand and the TLRs as receptors with default settings. Afterward, based on the best docking pose, a docked complex was selected for visualization and downstream processing. Further, their binding energy (ΔG) was calculated using the PRODIGY web server (https://wenmr.science.uu.nl/prodigy/). Subsequently, the interacting residues between *Ld*MAPV and TLRs were examined through PDBsum. After analysis, apo and complex forms of *Ld*MAPV were subjected to MD simulations of 100 ns, followed by the enumeration of RMSD, RMSF, Rg, solvent accessible surface area (SASA), and hydrogen bonds. Furthermore, simulation trajectories were examined and visualized at every 25 ns time interval through Pymol (https://pymol.org/2/). In addition, the secondary structural changes in the *Ld*MAPV model were analyzed using the Define Secondary Structure of Protein (DSSP) program (31).

### 2.10 Normal mode analysis of docking complex

The iMODS server (https://imods.iqfr.csic.es/) was utilized to evaluate the deformability and residue mobility in the protein structure. To assess the protein stability, iMODS uses normal mode analysis (NMA) to calculate the internal dihedral coordinates. The output deformability plot indicates protein flexibility, while the B-factor demonstrates atomic deformation from its equilibrium structure. The eigenvalue represents the rigidity of molecular motion that defines the stability of the protein, and a high score denotes much harder distortion.

### 2.11 Immune simulation of the designed vaccine candidate

The *in silico* immune response generation by *Ld*MAPV was predicted using the C-ImmSim server (https://150.146.2.1/C-IMMSIM/index.php?page=1). This server utilizes the Celada-Seiden model to define the humoral as well as cell-mediated immune response of a mammalian immune system against a constructed vaccine. The immune simulation was performed with default parameters, including a random seed of 12345, a simulation volume of 10 µl, simulation steps of 100, and the vaccine injection without LPS. Simultaneously, the obtained result was compared to immune simulation data for *L. chagasi* A2 immunogenic protein (Accession No. GQ290460) as a reference.

### 2.12 Gene synthesis, cloning and purification

Java Codon Adaptation Tool (JCat) (http://www.jcat.de) was employed to obtain optimized codons along with its codon adaptation index (CAI) value and GC content for the determination of optimal expression level in the *E. coli* K-12 strain. The codon-optimized sequence of the designed multi-epitope vaccine was synthesized from GenScript Biotech Co. into the pUC57 cloning vector. The construct was digested with *Bam*HI and *Hind*III and then inserted into the pET-28a expression vector using the same restriction sites. The recombinant plasmid was then transformed into BL21(DE3) pLys strain and cultured in LB broth supplemented with kanamycin (50 μg/ml) and chloramphenicol (34 μg/ml) at 37°C overnight in a shaking incubator. After transferring the 1% starter culture to LB broth with appropriate antibiotics, the culture was incubated at 37°C until the OD_600nm_ reached 0.5–0.6, followed by induction using 0.1-1 mM IPTG for 4 hours at 37°C. The induced cells were centrifuged, and then the pellet was resuspended in lysis buffer [50 mM HEPES pH 7.0, 300 mM KCl, 2 mM β-mercaptoethanol (β-ME), 0.5% N-lauroylsarcosine, 1 mM phenylmethylsulfonyl fluoride (PMSF), and 25 mM imidazole], and sonicated for 15 min with a cycle of 10 s pulse and 20 s pause. The sonicated sample was centrifuged, filtered, and kept for binding to pre-equilibrated Ni-NTA beads at 4°C for 1 hour. The column was washed with 30-75 mM imidazole to remove non-specific proteins, and the desired protein was eluted with 150-500 mM imidazole. The protein purity was examined on 12% SDS-PAGE, whereas the protein concentration was estimated using NanoDrop 2000c. Furthermore, western blotting was performed to confirm the band of vaccine protein observed on SDS-PAGE. The bands were electro-transferred onto nitrocellulose membrane, and then the membrane was blocked using 5% (w/v) skim milk in 1X TBS containing 0.1% Tween-20 (TBST) for 1 hour at room temperature, followed by washing with 1X TBST. Next, the membrane was incubated with HRP-conjugated His-tag monoclonal antibody (Cell Signalling Technology) at 1:5000 dilution for 1 hour at room temperature. After washing three times with 1X TBST, bands were visualized employing ECL Prime Detection Reagent (GE Healthcare) following the manufacturer’s instructions.

### 2.13 Mouse and human cell lines

The RAW264.7 murine macrophage and THP-1 human monocyte cell lines were kind gifts from Dr. Nooruddin Khan (University of Hyderabad, Hyderabad, India). RAW264.7 and THP-1 monocytes were distinctly grown in Dulbecco’s Modified Eagle Medium (DMEM) and Rosewell Park Memorial Institute (RPMI)-1640 with penicillin-streptomycin and 10% Fetal Bovine Serum (FBS). These cell lines were maintained in an incubator at a temperature of 37°C and 5% CO_2_.

### 2.14 Assessment of cytotoxicity and NO production

The purified *Ld*MAPV was treated with polymyxin B to chelate LPS contamination. THP-1 monocytes were incubated with 10 ng/ml of Phorbol 12-Myriatate 13-Acetate (PMA, Sigma-Aldrich) for 12 hours, followed by 24 hours in fresh RPMI media. Macrophages were seeded in a 96-well culture plate, and various concentrations (0.5, 1, 2, 5, 10, and 20 µg/ml) of purified vaccine along with 1 µg/ml of lipopolysaccharide (LPS) and 10 µg/ml of *L. donovani* 6-phosphogluconate dehydrogenase (*Ld*6PGDH) were added and kept for 24 and 48 hours. Subsequently, the MTT assay was performed to assess the cytotoxic effect of *Ld*MAPV on macrophages, as described earlier (32). Further, the level of nitric oxide (NO) in murine macrophages (RAW264.7) upon treatment of *Ld*MAPV was estimated utilizing the Griess reagent, as mentioned previously (33) with slight alterations. The absorbance was measured at 570 nm, and the nitrite content of the samples was calculated employing a standard curve of known concentrations (0-100 µM) of sodium nitrite.

### 2.15 Measurement of *Ld*MAPV induced cytokines

RAW264.7 and THP-1 macrophages (1×10^5^ cells per well) were seeded in a flat-bottom 96-well culture plate, and culture supernatant was collected at 24 and 48 hours after stimulation with different concentrations of *Ld*MAPV, *Ld*6PGDH, and lipopolysaccharide. Various cytokines, including TNF-α, IL-1β, IL-6, IL-10, and IL-12, were assessed through sandwich ELISA following the manufacturer’s instructions (BD Pharmingen). Briefly, 96-well plates were coated with the respective primary antibodies and incubated at 4°C overnight. Thereafter, plates were gently washed using Phosphate Buffer Saline with 0.5% Tween-20 (PBST) for 4-5 times, followed by incubation with 10% FBS for 1 hour to prevent non-specific interactions. Additionally, corresponding standards of cytokines were diluted in the range of 0.3-2.0 ng/ml and dispensed to the corresponding plate. Concurrently, the culture supernatant of vaccine-treated macrophages was also added and incubated at 4°C overnight. Next, plates were washed, and horseradish peroxidase (HRP)-conjugated specific secondary antibodies were added and kept at room temperature for 1 hour, followed by the addition of the substrate and incubation for 30 min. After that, plates were washed with PBST, succeeded by detection through the TMB substrate using a multimode reader (Tecan Spark) at a wavelength of 450 nm.

## 3 Results

### 3.1 Preliminary analysis of *Ld*MAcP sequence

The amino acid sequence of *Ld*MAcP was obtained from the UniProtKB database that contains 315 amino acids with a molecular weight of 35.17 kDa. Notably, the result of BLASTp suggested no significant homology with human proteins. Concurrently, the TMHMM v2.0 server projected the location of this protein outside of the cell membrane, which makes it easily accessible to humoral and cellular immune responses. Furthermore, the antigenicity analysis showed it as a probable antigen with a value of 0.5308, and AllerTOP v.2.0 projected it to be a non-allergen.

### 3.2 Prediction and successive selection of cytotoxic T-lymphocyte epitopes

CTL epitopes were predicted from *Ld*MAcP protein using a two-stage screening process. In total, 146 CTL epitopes (9-mer) were identified from all the 12 supertypes of Class I MHC using the NetCTL1.2 server. Subsequent evaluation revealed that only 24 epitopes were antigenic, immunogenic, and non-toxic, which was further reduced to 13 by analyzing their non-allergic potential. Afterward, these epitopes were evaluated based on better binding affinities towards human alleles, and epitopes interacting with at least 3 MHC-I alleles were considered for downstream processing. Among these epitopes, only six epitopes (ATAFLRGLF, FQDDYFYPV, RVLAAALLV, LYAALNPVI, IRVLAAALL, and GRLDNATNL) showed the desired binding affinity with the human alleles (**Table 1**). Finally, only four epitopes (ATAFLRGLF, FQDDYFYPV, RVLAAALLV, and GRLDNATNL) were selected for vaccine construction as LYAALNPVI overlapped with one of the chosen helper T-cell epitopes. The IRVLAAALL epitope was not preferred for vaccine construction because it coincided with another epitope (RVLAAALLV) and also covered a lesser population due to binding with fewer alleles.

**Table 1:** Prediction of Cytotoxic T-lymphocyte (CTL) epitopes from *Ld*MAcP.

CTL epitopes	Antigenicity	Allergenicity	Immunogenicity	Binding allele	Toxicity
ATAFLRGLF	Antigen	Non-allergen	0.19054	HLA-A*26:01 HLA-A*32:01 HLA-A*30:02	Non-Toxic
FQDDYFYPV	Antigen	Non-allergen	0.12652	HLA-A*02:06 HLA-A*02:01 HLA-B*39:01 HLA-C*08:02 HLA-C*12:03 HLA-A*01:01	Non-Toxic
RVLAAALLV	Antigen	Non-allergen	0.09486	HLA-A*02:06 HLA-C*15:02 HLA-E*01:01 HLA-A*30:01	Non-toxic
LYAALNPVI	Antigen	Non-allergen	0.04994	HLA-A*24:02 HLA-A*23:01 HLA-C*14:02	Non-toxic
IRVLAAALL	Antigen	Non-allergen	0.10371	HLA-B*27:05 HLA-B*39:01 HLA-B*38:01	Non-toxic
GRLDNATNL	Antigen	Non-allergen	0.07823	HLA-B*27:05 HLA-C*06:02 HLA-B*38:01	Non-toxic

### 3.3 Helper T-lymphocyte prediction and selection of IFN-γ inducing epitopes

In total, seventy-nine unique 15-mer-long HTL epitopes were identified with their respective MHC-II binding molecules, of which 17 were found to be non-allergenic, antigenic, and non-toxic. Out of 17 epitopes, 9 HTL epitopes showed IFN-γ cytokine eliciting ability, and subsequently, four epitopes (NPALYAALNPVIDEH, HTQRTIQSATAFLRG, LVAAAVSVDARLVVR, and YNSSLVYTRSTHTQR) were chosen for vaccine construction (**Table 2**). The epitopes LVAAAVSVDARLVVR and YNSSLVYTRSTHTQR were selectedfrom the respective overlapping HTL epitopes, AAAVSVDARLVVRMV and SSLVYTRSTHTQRTI, as the preferred epitopes provided higher antigenicity to the proposed vaccine candidate as compared to other screened HTL epitopes. However, ASKLIRVLAAALLVA, SKLIRVLAAALLVAA, and MASKLIRVLAAALLV were not considered for vaccine construction as they overlapped with the selected CTL epitope (RVLAAALLV).

**Table 2:** Helper T-lymphocyte (HTL) epitopes prediction from *Ld*MAcP.

HTL epitopes	Antigenicity	Allergenicity	Binding allele	IFN-γ inducer	Toxicity
NPALYAALNPVIDEH	Antigen	Non-allergen	HLA-DRB1*10:01	Positive	Non- toxic
AAAVSVDARLVVRMV	Antigen	Non-allergen	HLA-DRB1*03:01	Positive	Non- toxic
HTQRTIQSATAFLRG	Antigen	Non-allergen	HLA-DRB5*01:01	Positive	Non- toxic
LVAAAVSVDARLVVR	Antigen	Non-allergen	HLA-DRB1*03:01	Positive	Non- toxic
ASKLIRVLAAALLVA	Antigen	Non-allergen	HLA-DRB1*15:01	Positive	Non- toxic
SKLIRVLAAALLVAA	Antigen	Non-allergen	HLA-DRB1*15:01	Positive	Non- toxic
MASKLIRVLAAALLV	Antigen	Non-allergen	HLA-DRB1*15:01	Positive	Non- toxic
SSLVYTRSTHTQRTI	Antigen	Non-allergen	HLA-DRB1*07:01	Positive	Non-toxic
YNSSLVYTRSTHTQR	Antigen	Non-allergen	HLA-DRB1*07:01	Positive	Non-toxic

### 3.4 Linear B-lymphocyte prediction

**Figure 2:**
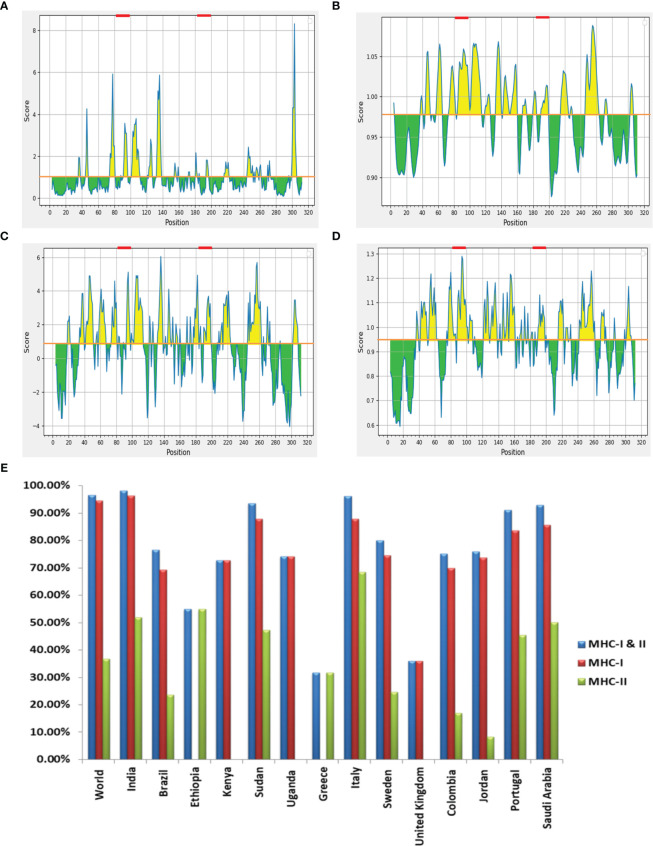
**(A)** Emini surface accessibility, **(B)** Karplus and Schulz flexibility, **(C)** Parker hydrophilicity and **(D)** Chou and Fasman beta-turn prediction of selected LBL epitopes, where X- and Y-axis represent the respective sequence position and scores. The orange line denotes the average values of calculated scores and the yellow regions above the average values are considered as good, while the selected LBL epitopes are marked as red. **(E)** Worldwide population coverage by T-cell epitopes of multi-epitope vaccine with their MHC-I (Red), MHC-II (Green) and combined MHC-I & II (Blue) coverage rate.

The LBL epitopes from *Ld*MAcP were predicted through the ABCpred server, and 28 (16-mer) epitopes were found with a score of 0.5 or higher. Subsequent analysis with BepiPred assisted in the selection of 14 epitopes that were common on both servers. Further studies revealed that only three epitopes possessed exomembranic, antigenic, and non-allergenic properties (**Table 3**). Based on the predicted surface accessibility, hydrophilicity, flexibility, and beta-turn of these three epitopes, two of them (AWIEGLCTDFNARTSC and LSLVESPLFPSTQYNS) had all of the desired properties by exceeding their respective threshold values of 1.0, 0.819, 0.977, and 0.944 (**Figures 2A–D**). Thus, the presence of two linear B-cell epitopes, “AWIEGLCTDFNARTSC” and “LSLVESPLFPSTQYNS,” in the vaccine protein could help to generate the strong neutralizing antibodies against the leishmanial parasites.

**Table 3:** Assessment of linear B-lymphocyte (LBL) epitopes from *Ld*MAcP.

B-cell epitopes	Bepi 2	Transmembrane topology	Antigenicity	Allergenicity	Toxicity
AWIEGLCTDFNARTSC	Yes	Outside	Antigen	Non-allergen	Non-toxic
LSLVESPLFPSTQYNS	Yes	Outside	Antigen	Non-allergen	Non-toxic
ALLVAAAVSVDARLVV	No	Outside	Antigen	Non-allergen	Non-toxic

### 3.5 Similarity and population coverage analysis of selected epitopes

In order to prevent auto-immunity, the selected T- and B-cell epitopes on the designed vaccine should not display any similarity with human proteins (34); hence cross-reaction evaluation is an important feature to be considered during vaccine designing. Cross-reactive analysis of epitopes in reference to human proteome delineated that all the preferred epitopes didn’t show significant homology with host proteins, advocating no cross-reactivity for normal human cells. Coverage of the maximum allele population makes a vaccine candidate more effective, so population coverage was estimated for the chosen T-cell epitopes and their corresponding HLA alleles. When HTL and CTL epitopes were put together, they covered 96.56% of the world’s population. India had the highest coverage at 98.27%, ahead of Italy (96.15%) and Sudan (93.63%) (**Figure 2E**). The findings firmly established that the proposed vaccine construct could aid in the fight against VL in most of the affected countries worldwide.

### 3.6 Multi-epitope vaccine construction and characterization

**Figure 3:**
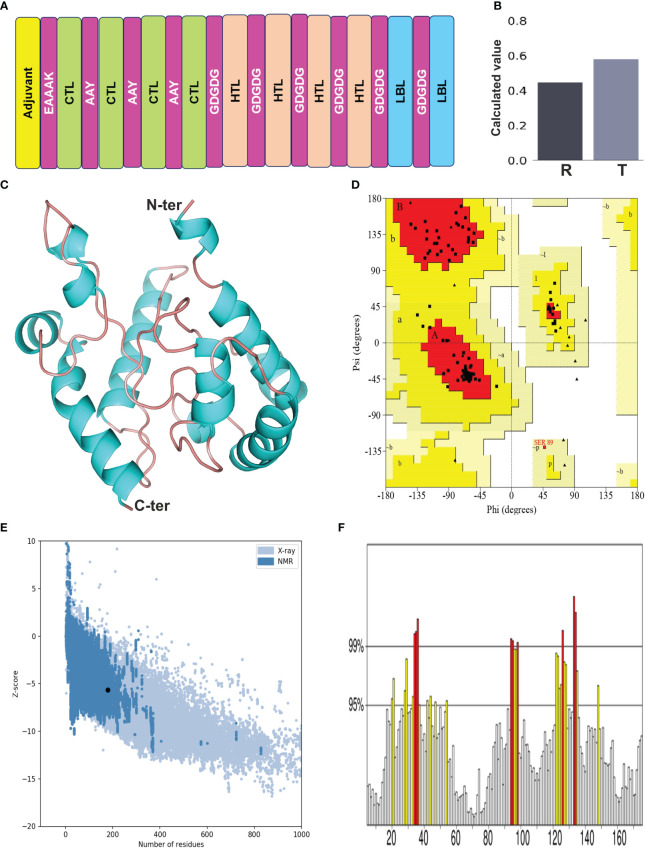
**(A)** Designing of multi-epitope vaccine from *Ld*MAcP. The corresponding CTL, HTL and B-cell epitopes are presented as box with green, beige and blue color, while adjuvant and linkers are represented in yellow and magenta color, respectively. **(B)** Solubility assessment of vaccine construct using Protein-Sol, where R and T correspondingly delineate the reference protein and designed vaccine protein. **(C)** Modelled three-dimensional structure of the multi-epitope vaccine showing α-helices (cyan) and random coils (beige). **(D)** Ramachandran plot indicating the presence of amino acids in favoured, allowed and disallowed region. **(E)** ProSA-web validation presenting Z-score of predicted 3D structure. **(F)** ERRAT plot of the vaccine construct.

The selected epitopes were joined employing particular linker sequences to design a vaccine construct (*Ld*MAPV). In total, the final vaccine construct consisted of 179 amino acids encompassing 10 epitopes (4 CTL, 4 HTL, and 2 LBL) that were combined through AAY and GDGDG linkers. The AAY linker enhances immunogenicity of the multi-epitope vaccine (35), while the GDGDG linker aids in the expression of the multi-epitope vaccine by virtue of their flexible (Gly) and hydrophilic (Asp) amino acid composition (36). The administration of TLR agonists as adjuvants within vaccine candidates are reported to induce strong T-cell and antibody-mediated responses (37), hence the N-terminus was appended with a TLR-4 agonist as adjuvant, i.e., RS-09 (APPHALS), using a rigid linker (EAAAK). This linker maintains an optimal distance between functional components of the vaccine to improve stability and preserve their unique function. Moreover, when incorporating an adjuvant, it’s advisable to position the EAAAK linker right after the adjuvant sequence (38). Altogether, the designed vaccine comprised of an adjuvant, followed by 4 CTL, 4 HTL, and 2 B-cell epitopes (**Figure 3A**). The antigenicity analysis of *Ld*MAPV exhibited it as highly antigenic with a score of 0.9551 on the VaxiJen v2.0 server, whereas it was also found to be non-allergenic on both servers (AllerTOP v.2 and AllergenFP). Simultaneously, other physiochemical characteristics of *Ld*MAPV were also assessed using Expasy ProtParam. The molecular weight of *Ld*MAPV was enumerated to be 18.6 kDa, possessing a chemical formula of C_817_H_1243_N_231_O_267_S_2_. It consisted of 22 negatively and 11 positively charged residues with a theoretical pI of 4.46, indicating its negatively charged nature under physiological conditions. The respective computed instability and aliphatic index were observed to be 30.33 and 79.22, which classified this construct as a stable and highly thermostable protein. The estimated half-life was 4.4 hours *in vitro*, while it was greater than 20 and 10 hours in yeast and *Escherichia coli*, respectively, highlighting its stability in the *in vivo* system. Additionally, the GRAVY value of *Ld*MAPV was observed to be −0.149 that delineates the vaccine as hydrophilic in nature and having better contacts with water molecules. The predicted solubility scores of this construct by Protein-sol and SOLPro were 0.58 (**Figure 3B**) and 0.95, respectively (**Table 4**), which revealed its solubility when overexpressed in the *E. coli* system. Furthermore, RaptorX solvent accessibility analysis revealed that 35% of residues were exposed, 32% were medium exposed, and 33% were buried. However, only some residues (4%) were observed in the disordered domains.

**Table 4:** Physicochemical parameters of the multi-epitope vaccine construct.

Parameter	Value	Comments
Number of amino acids	179	Suitable
Molecular weight	18.6 kDa	Average
Theoretical pI	4.46	Acidic
Instability index	30.33	Stable
Aliphatic index	79.22	Thermo-stable
GRAVY	−0.149	Hydrophilic
Solubility	0.58 and 0.95	Soluble
Estimated half-life in yeast	>20 hrs	Satisfactory
Estimated half-life in *E.coli*	>10 hrs	Satisfactory

### 3.7 Structural features of the designed vaccine candidate

The secondary structure of *Ld*MAPV was predicted using PSIPRED v4.0 and the PDBsum online server. PSIPRED v4.0 suggested the presence of 43.02% alpha-helix, 4.47% extended strands, and 52.51% random coils (**Figure S1A**). It also contains 44.13% nonpolar residues, 27.37% polar, 17.88% hydrophobic, and 10.62% aromatic plus cysteine (**Figure S1B**). On the other hand, the PDBsum web server that predicts secondary structural content based on the 3D structure of proteins revealed that 45.81% and 51.96% of residues participate in the construction of the corresponding alpha helix and random coil, while only 2.23% formed the beta-strand (**Table 5**). Using the top ten threading templates that exhibited alignment to the primary sequence of *Ld*MAPV, the top five 3D structures of *L*dMAPV were generated by the I-TASSER web server. The confidence score (C-score) is an essential factor to measure the quality of a model, and its higher value ensures a better quality of the generated structure. The C-score values for five predicted models were in the range of -5 to -3.49, wherein the model with a C-score of -4.94 was chosen for further refinement due to the presence of lower proportion of residues in the disallowed region of the Ramachandran plot. The generated vaccine structure was refined using the GalaxyRefine server (**Figure 3C**), followed by validation through the Ramachandran plot, which displayed 90.5%, 8.8%, and 0.7% in the most favored, additionally allowed, and generously allowed regions, respectively. Notably, no residues were observed in the disallowed region (**Figure 3D**). Furthermore, ProSA-web and ERRAT servers were utilized to determine the quality and inevitable potential errors, respectively. ProSA-web enumerated a Z-score of -5.96 for the refined 3D model (**Figure 3E**), which falls in the range of generally found native proteins of similar size. It also showed an overall quality factor of 83.04% in the ERRAT server (**Figure 3F**) that altogether validated the refined *Ld*MAPV model.

**Table 5:** The secondary structural contents of the vaccine construct.

Features	PsiPred server		PDBSum	
	Amino acids	Percentage	Amino acids	Percentage
Alpha-helix	77	43.02	82	45.81
Beta strand	8	4.47	4	2.23
Random coil	94	52.51	93	51.96

### 3.8 Screening of discontinuous B-cell epitopes

A total of four discontinuous (conformational) B-cell epitopes of various lengths were obtained from the validated 3D structure of *Ld*MAPV with acceptable scores. The top-scoring epitope was seven amino acids long containing the residues G98, D99, G100, D101, G102, H103, and Q105. It had a Protrusion Index (PI) value of 0.72, which refers to the presence of 72% residues in the ellipsoid region (**Table 6**).

**Table 6:** Conformational B-cell epitopes from vaccine protein using Ellipro server.

Residue	No. of residues	PI value
G98, D99, G100, D101, G102, H103, Q105	7	0.72
R18, G19, L20, F21, A22, A23, Y24, F25, Q26, D27, D28, Y29, F30, A40, R50, L51, D52, N53, A54, N56, L57, G58, D59, G60, D61, G62, N63, P64, A65, L66, A68, A69, N71, P72, D75, E76, G78	37	0.696
A1, P2, P3, H4, A5, L6, S7, E8, Y36	9	0.673
R116, G117, G118, D119, G120, D121, G122, Y123, N124, R131, S132, T133, H134, T135, Q136, R137, G138, D139, G140, D141, G142, A143, W144, I145, C149, T150, D151, F152, N153, A154, R155, T156, S157, C158, G159, D160, G161, D162, L164, V167, E168, L171, F172, S174, T175, Y177, N178, S179	48	0.667

### 3.9 *In silico* disulfide engineering for vaccine stability

**Figure 4:**
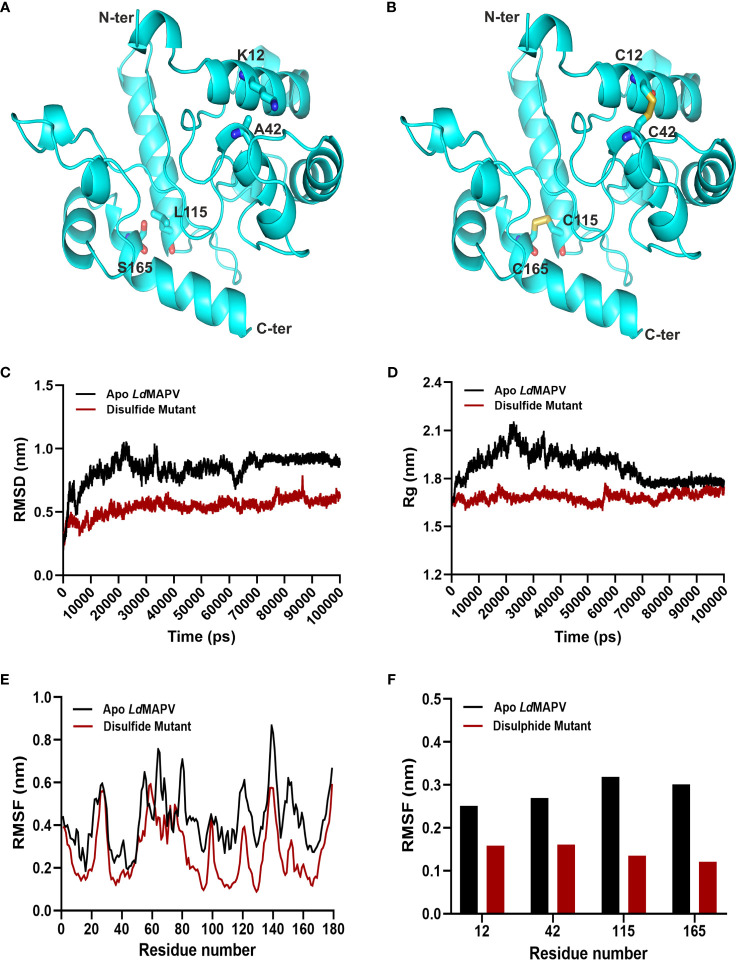
Disulfide engineering of *Ld*MAPV. **(A)** Multi-epitope vaccine structure showing residues plausible for disulfide formation. **(B)** Disulfide-engineered vaccine structure displaying disulfide bonds between mutated residues. **(C)** RMSD, **(D)** Rg and **(E)** RMSF plots for apo *Ld*MAPV and disulphide mutant protein after 100 ns of simulation. **(F)** Comparison of RMSF graph for residues mutated for disulfide engineering.

Twenty-one pairs of amino acids were observed to be capable of forming a disulfide bond during the disulfide engineering of *Ld*MAPV. Nevertheless, only two pairs of amino acids were ultimately chosen after analyzing the χ^3^ angle and the energy score, as their values fulfilled the requisite. Therefore, four mutations on the residue pairs Lys12 - Ala42 and Leu115 - Ser165 were generated (**Figures 4A, B**) with χ^3^ angles of -81.74 and -70.83 and energy scores of 1.79 and 2.17 kcal/mol, respectively. Additionally, the antigenic and allergenic properties of disulfide engineered *Ld*MAPV were also assessed and compared with the apo vaccine (**Table S1**). An antigenicity of 0.9658 and a non-allergenic nature were found for the disulfide-engineered *Ld*MAPV. Moreover, Protein-Sol and SOLpro projected a respective solubility score of 0.591 and 0.9593 for the mutated *Ld*MAPV. These outcomes suggested a comparatively more antigenic and soluble nature of mutated *Ld*MAPV than its apo form. In addition, molecular dynamic simulations were carried out for the apo and mutated vaccine proteins. Comparison of RMSD data revealed that the respective average RMSD values for *Ld*MAPV in its apo and mutated forms were 0.84 and 0.54 nm (**Figure 4C**), suggesting the mutated form of the vaccine to be significantly more stable than the apo form. Further, the protein structure compactness was analyzed using the Rg graph (**Figure 4D**) and the mutated form was found more compact than the apo one, with an average Rg value of 1.68 nm. The root mean square fluctuation (RMSF) of individual residues was also determined in each case to examine residual mobility (**Figures 4E, F**). The mean RMSF values of backbone atoms for the apo and mutated forms were 0.42 and 0.27 nm, respectively, showing reduction in backbone fluctuation among the residues of the disulfide mutated form compared to the apo *Ld*MAPV.

### 3.10 Molecular docking of *Ld*MAPV with TLR receptors

**Figure 5:**
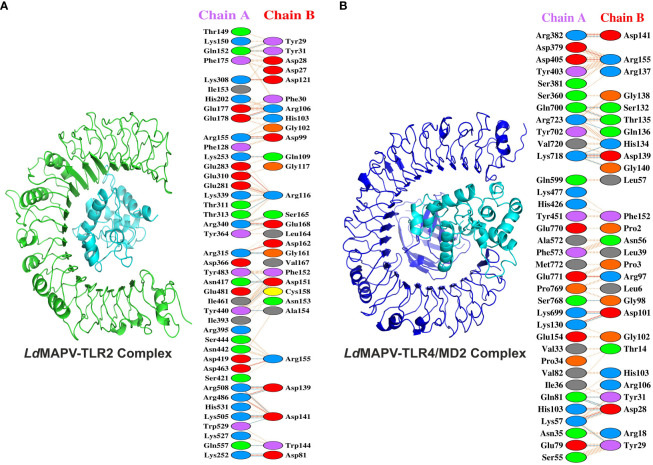
Docking studies of vaccine protein and human toll-like receptors. **(A)** *Ld*MAPV-TLR2 and **(B)** *Ld*MAPV-TLR4/MD2 complexes reveal numerous non-bonded contacts, hydrogen bonds and salt bridges. The structures of *Ld*MAPV, TLR2 and TLR4/MD2 are in cyan, green and blue cartoon representation, respectively.

Toll-like receptors (TLRs) are known to localize on the cell surface and induce a cascade of immune responses when activated by the vaccine. Hence, molecular docking was carried out to investigate the binding affinity between the designed *Ld*MAPV and TLRs (TLR2 and TLR4/MD2). Out of the resulting 30 clusters, the one that was docked into the receptor cavity with the lowest energy score was subjected to downstream processing. The lowest energy score of the *Ld*MAPV-TLR2 complex was -875.7, while it was -891.3 for the *Ld*MAPV-TLR4/MD2 complex. The number of residues involved at the complex interface for *Ld*MAPV and TLR2 was 29 and 42, respectively, while their corresponding interface areas were 1827 and 1690 Å^2^. The binding force analysis identified 304 nonbonded contacts, 27 hydrogen bonds, and 15 salt bridges in the *Ld*MAPV-TLR2 complex (**Figure 5A**). Concurrently, the total corresponding residues observed for *Ld*MAPV and TLR4/MD2 interface were 28 and 35 in the *Ld*MAPV-TLR4/MD2 complex. Among the 35 residues, 11 (Lys699, Gln700, Tyr702, Lys718, Val720, Arg723, Ser768, Pro769, Glu770, Glu771, and Met772) were from the MD2 co-receptor. The interface areas possessed by *Ld*MAPV and TLR4/MD2 were 1844 and 1737 Å^2^, respectively, whereas the complex contained 199 nonbonded contacts, 9 salt bridges, and 19 hydrogen bonds (**Figure 5B**). These findings highlighted the proposed vaccine candidate’s strong affinity for human toll-like receptors. Further, the selected docked conformations were used for the enumeration of binding affinity or Gibbs free energy (ΔG) and dissociation constant (K_d_). At 25°C, the ΔG values for *Ld*MAPV-TLR2 and *Ld*MAPV-TLR4/MD2 complexes were computed to be -19 and -15.2 kcal/mol with K_d_ values of 1.2 × 10^-14^ and 6.8 × 10^-12^ M, respectively (**Table 7**). Simultaneously, the number of interfacial contacts (ICs) per property was calculated within the default distance of 5.5 Å. There were 36 charged-charged, 0 polar-polar, and 20 apolar-apolar interfacial contacts observed in the *Ld*MAPV-TLR2 complex, while for the *Ld*MAPV-TLR4/MD2 complex, the corresponding charged-charged, polar-polar, and apolar-apolar ICs were enumerated as 29, 7, and 25, suggesting the hydrophilic nature of most of the interfacial contacts.

**Table 7:** Binding affinity analysis of the vaccine-receptor complexes.

Vaccine-receptor complex	Center energy score	Lowest energy score	Binding affinity (ΔG)	Dissociation constant (Kd)
*Ld*MAPV-TLR2	-854.5	-875.7	-19.0 kcal/mol	1.2 × 10^-14^ M
*Ld*MAPV-TLR4/MD2	-770.9	-891.3	– 15.2 kcal/mol	6.8 × 10^-12^ M

### 3.11 Conformational stability of docked complexes by MD simulation

**Figure 6:**
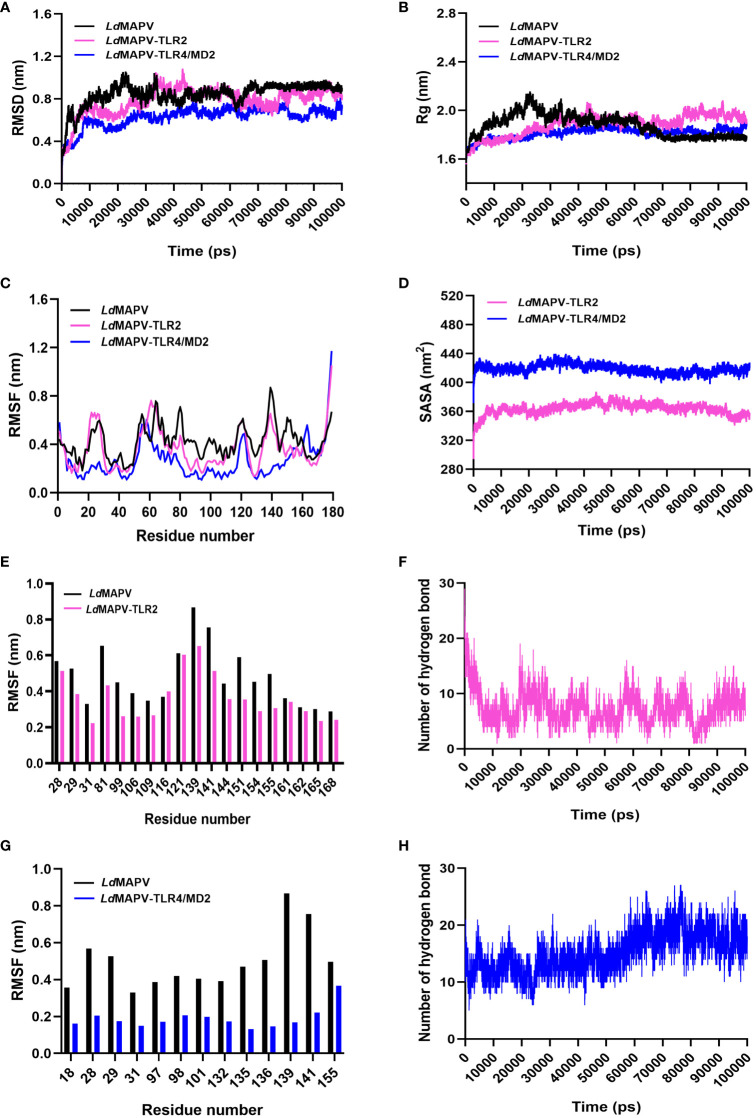
Comparative molecular dynamics simulations. **(A)** RMSD of backbone atoms, **(B)** R_g_ and **(C)** RMSF plots of *Ld*MAPV in apo and complex forms. **(D)** SASA analysis for *Ld*MAPV complexed with TLRs. **(E, G)** RMSF comparison of complex forming residues of *Ld*MAPV with TLRs. **(F, H)** Plots showing number of hydrogen bonds of *Ld*MAPV-TLR2 and *Ld*MAPV-TLR4/MD2 complexes during dynamics.

The stability of *Ld*MAPV structure before and after the establishment of contact with TLR2 and TLR4/MD2 was analyzed through the comparison of RMSD, Rg, RMSF, H-bond, and SASA of *Ld*MAPV in apo and complex forms (**Table S2**). The average RMSD of *Ld*MAPV in the apo form and complex with TLR2 and TLR4/MD2 was 0.842, 0.78, and 0.628 nm, respectively. As evident from the plot, the complex form of *Ld*MAPV attained a lower deviation value than the apo form, inferring that the complexes were structurally more stable than the apo form (**Figure 6A**). Succeedingly, the compactness of *Ld*MAPV structure was analyzed using the Rg graph, where apo *Ld*MAPV and its complex states (*Ld*MAPV-TLR2 and *Ld*MAPV-TLR4/MD2) were found to be stable with no obvious structural expansion or contraction, with corresponding average Rg values of 1.874, 1.885, and 1.812. The least and steadiest Rg was observed for the *Ld*MAPV-TLR4/MD2 complex that suggests more compactness of protein inside the TLR4/MD2 receptor pocket (**Figure 6B**). Although the Rg mean value for vaccine protein in the *Ld*MAPV-TLR2 complex was comparatively higher, it remained stable (ranging from 1.55 to 2.03 nm) throughout the simulation period. The RMSF graph was also employed in order to assess the flexibility of amino acids in a protein structure. The average RMSF values for apo *Ld*MAPV and its complex with TLR2 and TLR4/MD2 were 0.42, 0.36, and 0.26 nm, respectively, which indicated fluctuation of vaccine structure in the complex states was lower than the free-state (**Figure 6C**). The interacting vaccine residues with the receptor molecule displayed a lower RMSF value (**Figures 6E, G**) that signified the vaccine-TLR interface stability during the dynamics.

Moreover, the stability of complexes was evaluated by enumerating the hydrogen bonds between receptor and ligand molecules. The average number of hydrogen bonds was found to be 7 and 15 in the vaccine complexes with TLR2 and TLR4/MD2 receptors, respectively. Also, *Ld*MAPV-TLR4/MD2 had the most hydrogen bonds that stayed in place during dynamics (**Figures 6F, H**) and made a big difference in the close binding of the vaccine to the TLR4/MD2 receptor. In addition, the calculation of SASA is widely used to evaluate the alteration in surface accessibility of a complex to solvent molecules, and the higher value of SASA represents the expansion of the protein backbone. The respective average SASA values for *Ld*MAPV-TLR2 and *Ld*MAPV-TLR4/MD2 complexes were 363.59 and 418.84 nm^2^ (**Figure 6D**), suggesting more solvent to be accessed by the *Ld*MAPV-TLR4/MD2 complex.

### 3.12 Insights into key interactions during MD Simulations

To further check the constancy of the docked complex, snapshots were taken at different time frames during the MD simulations. Visualization of the MD trajectories revealed that the binding of receptor-ligand remained intact throughout the simulation period (**Figure S2**). The vaccine protein also displayed minimal structural changes in the complex forms, further supporting the consistency of complexes. The initial trajectory of the *Ld*MAPV-TLR2 complex exhibited several salt bridges and hydrogen bonds between vaccine protein and TLR2. Most of the hydrogen bonds were lost during the 25 ns simulation period except Glu178 and Arg340 of TLR2 and Arg106 and Glu168 of vaccine protein; however, new inter-chain hydrogen bonds were generated after 25 to 100 ns. Concurrently, among the 15 salt bridges of the initial frame, only three between Glu178, Arg340, and Arg486 of TLR2 and Arg106, Glu168, and Asp141 of vaccine remained stable for 25 ns simulation. Notably, the salt bridge between Glu178 of TLR2 and Arg106 of the vaccine remained stable until the simulation ended.

Similarly, the initial trajectory of the *Ld*MAPV-TLR4/MD2 complex delineated seventeen hydrogen bonds and seven salt bridges. The only interaction that lasted until the end of the simulation was between Arg382 of TLR4/MD2 and Asp141 of the vaccine. Out of 17 hydrogen bonds, only the one between Arg382 of TLR4/MD2 and Asp141 of vaccine continued to be stable till the end of simulation. However, another hydrogen bond between Thr37 of TLR4/MD2 and Asp99 of the vaccine protein present at the 25 ns trajectory was also consistently observed in the remaining period of simulation.

### 3.13 Secondary structure analysis of each simulation system

The secondary structural changes of proteins during MD simulation were observed through DSSP analysis, which exhibited that the secondary structures of *Ld*MAPV, including coil, helix, beta sheets, and turns, nearly stayed unchanged in both apo and complex forms with slight transitions only (**Figure S3**). Moreover, the vaccine structure, where most of the structural elements were in the helix (residue numbers 105 to 115), remained stable throughout the simulation. Comparative investigation indicated that the percentage of the random coil was slightly higher in the complex state of *Ld*MAPV than in the apo form. The average coil percentages for vaccine in apo, *Ld*MAPV-TLR2, and *Ld*MAPV-TLR4/MD2 complexes were 30.1, 31.21, and 32.65, respectively. However, the percentage of the alpha helix was lower in the complex than in its apo form, with corresponding average percentages of the helix of 37.59, 31.26, and 31.08 for the apo vaccine, *Ld*MAPV-TLR2, and *Ld*MAPV-TLR4/MD2 complex.

### 3.14 Normal mode analysis of the docked complexes

NMA was used to understand the motion and stability of residues in the protein structures. The peaks of the deformability plot were marked as the non-rigid portion of the protein. The deformability plot of the vaccine construct displayed some fluctuations, while very few fluctuations were observed in the *Ld*MAPV-TLR2 and *Ld*MAPV-TLR4/MD2 complexes (**Figures S4A, D, G**) that suggest the stabilization of the complexes. Additionally, the thermal motion of residues inside the protein is denoted by a B-factor value that corresponds to the root mean square fluctuation (RMSF). The computed B-factors of all the residues in the apo, *Ld*MAPV-TLR2, and *Ld*MAPV-TLR4/MD2 were below 1.0 (**Figures S4B, E, H**), implying stabilization of the protein structures. In addition, the eigenvalue of the *Ld*MAPV protein was 2.84924e^− 05^, whereas it was 7.268381e^− 05^ and 8.999161e^− 05^ for the *Ld*MAPV-TLR2 and *Ld*MAPV-TLR4/MD2 complex, respectively (**Figures S5C, F, I**). The higher eigenvalue for complex forms than the apo *Ld*MAPV indicated that much more energy is needed to deform its complexes.

### 3.15 *In silico* immunogenicity analysis of vaccine protein

**Figure 7:**
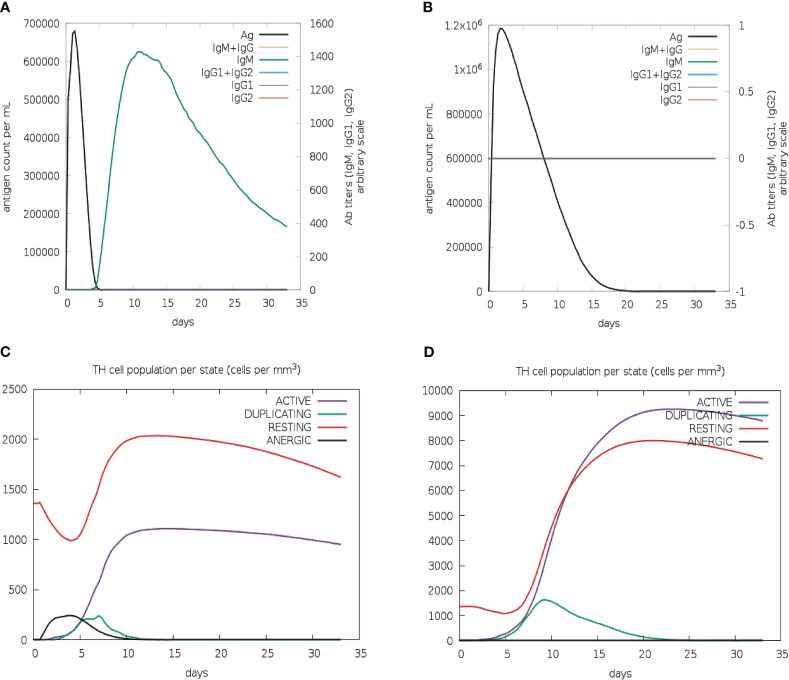
*In silico* immune simulation of multi-epitope vaccine. Levels of immunoglobulins and antigen for vaccine candidate **(A)** and *L. chagasi* A2 protein **(B)**. TH memory cell population for vaccine candidate **(C)** and A2 protein **(D)**.

To evaluate the effectiveness of the vaccine candidate, a comparative immunological simulation study was conducted using the proposed vaccine candidate and the previously reported *Leishmania chagasi* A2 protein sequence as a reference that is known to induce a potent Th1 immune response against VL (39). The analysis revealed that after administering the designed vaccine, the antigen concentration reached around 6.8× 10^6^/ml and dropped to zero on day five. After five days following the injection of the vaccine, a potent antibody response with IgM as the dominant immunoglobulin was seen, and after ten days of vaccination, IgM levels peaked at about 1400 on an arbitrary scale (**Figure 7A**). Simultaneously, a long-lasting active B-cell population with a peak value of 500 cells per mm^3^ was also noted (**Figure S5A**).

The active T-helper cell population started increasing after three days of vaccination and reached a maximum of 1000 cells per mm^3^ after ten days (**Figure 7C**). The population of activated cytotoxic-T lymphocytes was found to rise to more than 600 cells per mm^3^, which remained stable for the next 30 days (**Figure S5C**). As expected, the population of resting cytotoxic-T lymphocytes rapidly decreased after the first two days of the vaccine injection that is required to promote the immunological reaction. Following vaccination, high levels of IFN-gamma (over 400,000 ng/ml) and interleukin-2 (over 100,000 ng/ml) were also observed. Moreover, a greater diversity of immune responses was shown by a lower Simpson index value when compared to the A2 protein (**Figures S5E–F**). The increased macrophage activity (around 110 cells per mm^3^) was also detected to facilitate the antigen presentation (**Figure S5G**). The immunological profile of the A2 protein revealed neither an antibody response nor an activated cytotoxic-T cell population (**Figures 7B**, **S5D**); however, active B-cell and macrophage populations were quite similar to those of vaccine protein (**Figures S5B, H**). In addition, there was a slight upsurge in IFN-γ level (over 500,000 ng/ml) in comparison to vaccine protein (**Figure S5F**). Furthermore, the active T-helper cell population rapidly increased in A2 protein as compared to the vaccine protein (**Figure 7D**); however, both proteins displayed proportionally equal persistence of such populations.

### 3.16 Cloning and purification of vaccine construct

**Figure 8:**
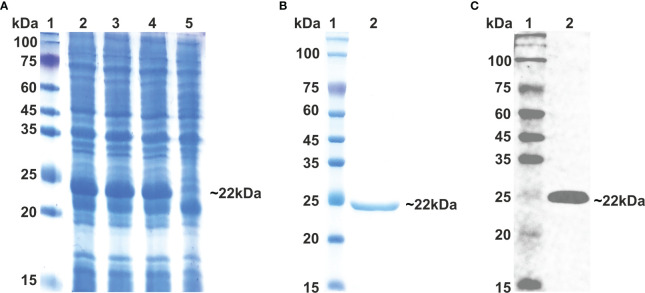
Expression and Purification of multi-epitope vaccine. **(A)** Expression of multi-epitope vaccine construct using different conc. of IPTG. Lane M indicates protein marker and Lanes 2-4 designate cells induced with 0.1, 0.5 and 1.0 0 mM of IPTG, while Lane 5 represents uninduced cells. **(B)** Purification of multi-epitope vaccine through affinity chromatography and **(C)** western blot analysis of purified vaccine protein. Lane 1 shows protein marker, whereas Lane 2 displays purified *Ld*MCPV.

As vaccine protein needs to be propagated into a bacterial system for optimal expression, the Java Codon Adaption tool (JCat) was used to optimize the codon of the vaccine construct sequence. The length of the optimized codon sequence was found to be 537 nucleotides, with a codon adaptation index (CAI) of 1.0 and GC content of 57.91%. The calculated CAI and GC content of the optimized sequence lie within the respective reference ranges of 0.8–1.0 and 30–70%, supporting the higher expression of the vaccine candidate in the bacterial host. The coding sequence of *Ld*MAPV was commercially synthesized, followed by sub-cloning into the pET-28a expression vector. The clone was confirmed through restriction digestion and DNA sequencing, which revealed no deletion or point mutation in the sequence. The confirmed clone was transformed into BL21(DE3) pLys cells, and then different concentrations of IPTG (0.1, 0.5, and 1 mM) were used to optimize the expression of the recombinant protein. Analysis of the resulting samples on SDS-PAGE revealed similar expression levels of the recombinant protein at all IPTG concentrations (**Figure 8A**). Subsequently, affinity chromatography was employed using a Ni^2+^–NTA column to purify the recombinant protein. Most of the contaminating proteins were washed away from the column with a buffer containing 30-75 mM imidazole, whereas vaccine protein was eluted between 150-500 mM imidazole concentration. Purified protein displayed a single band on SDS-PAGE with a molecular weight of approximately 22 kDa (**Figure 8B**), which corresponds to its projected molecular weight. The purity of the eluted protein was estimated to be about 95%, as observed on SDS-PAGE. Moreover, through western blot analysis, the identity of purified vaccine protein was confirmed as a band of approximately 22 kDa, devoid of any bacterial protein contaminants (**Figure 8C**). Finally, protein concentration was calculated by NanoDrop 2000c employing the molar extinction coefficient of 18,910 M^−1^ cm^−1^ and the molecular weight of 22,037 Da of *Ld*MAPV.

### 3.17 *Ld*MAPV stimulates nitric oxide production

The viability of *Ld*MAPV treated macrophages was measured by the reduction of MTT to formazan. The OD values of vaccine construct treated cells were observed to be comparable to those of the untreated cells (**Figures S6A–D**), delineating that protein didn’t affect the viability of mammalian macrophages even at the highest tested concentration, i.e., 20 µg/ml. Nitric oxide plays an essential function during the invasion of external proteins or pathogens inside the host cell and also activates inflammation and pathogenesis by removing the external stimuli or pathogen from the infection site. As the synthesis of nitric oxide is well defined in the murine macrophage RAW264.7, *Ld*MAPV was investigated for induction of NO production. RAW264.7 cells were treated with increasing concentrations of purified vaccine, and then culture supernatant was collected and subjected to assessment of NO using Griess reagent. Notably, *Ld*MAPV treated cells produced NO against all the tested concentrations in the range of 10–50 μM after 24 hrs, which is comparable to that of LPS treated macrophages, while untreated and *Ld*6PGDH treated cells showed negligible amounts (2–6 μM) of nitric oxide (**Figure S6E**). This outcome advocates for NO generation explicitly through *Ld*MAPV and its role in the defense mechanisms of host cells.

### 3.18 *Ld*MAPV elicits *in vitro* immune response

**Figure 9:**
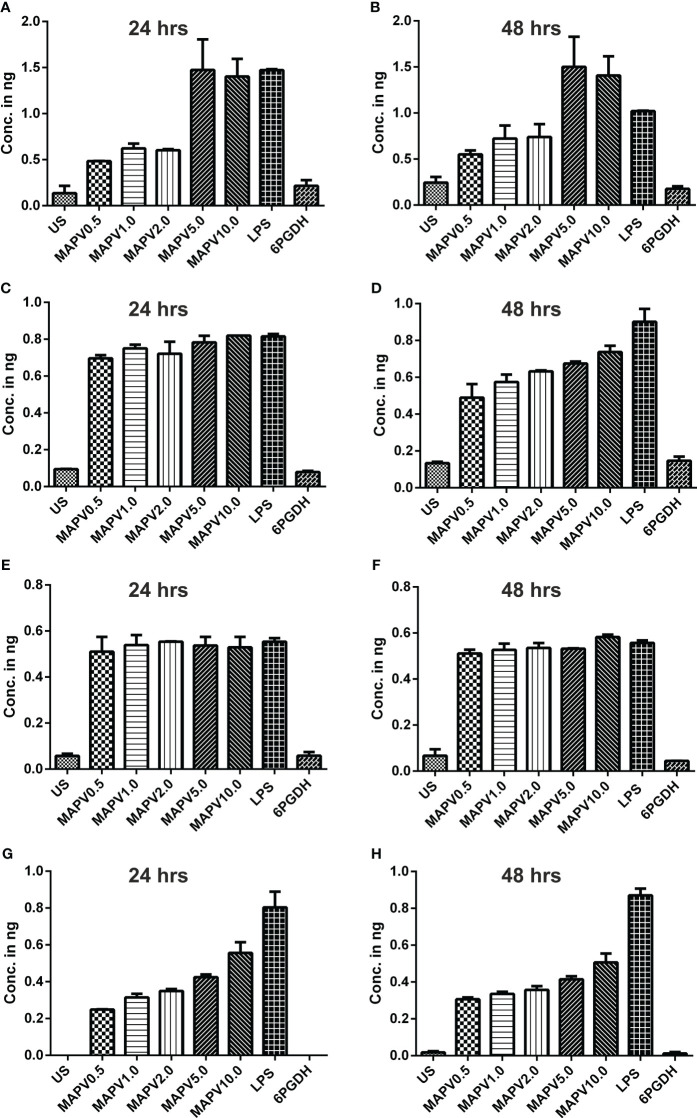
Upregulation of cytokines in murine macrophage. RAW 264.7 macrophages were treated with different concentrations (0.5, 1, 2, 5, and 10 µg/ml) of *Ld*MAPV for 24 and 48 hrs and then culture supernatant was assessed for TNF-α **(A, B)**, IL-1β **(C, D)**, IL-6 **(E, F)** and IL-10 **(G, H)** cytokine levels though sandwich ELISA. Untreated and *Ld*6PGDH treated cells were used as negative controls, whereas 1 µg/ml of LPS treated cells were taken as positive control. The p values for all the groups were significantly low (<0.05) in comparison to the negative controls. The experiments are representative of mean +/- SEM of two different experiments done in triplicates.

**Figure 10:**
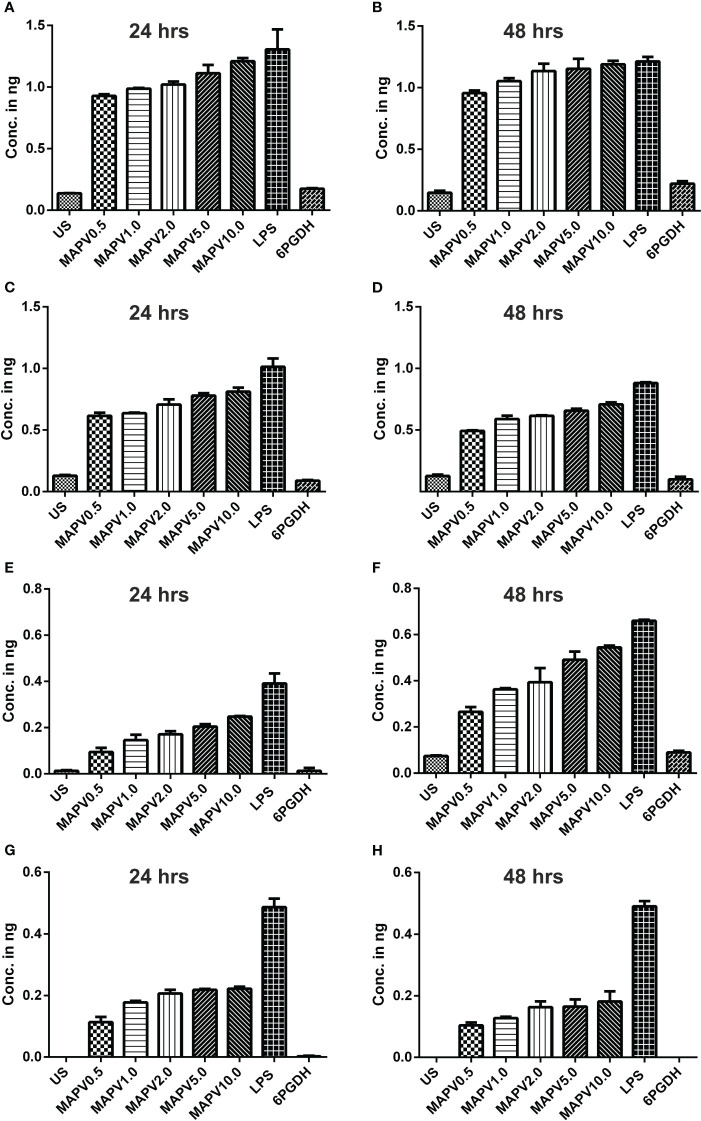
Cytokines elicitation by *Ld*MAPV in human macrophage. THP-1 macrophages were stimulated with different concentrations of *Ld*MAPV for 24 and 48 hrs and then culture supernatant was assessed for TNF-α **(A, B)**, IL-1β **(C, D)**, IL-6 **(E, F)** and IL-10 **(G, H)** cytokine levels. *Ld*6PGDH and LPS treated cells served as negative and positive controls. The p values for all the groups were low significantly (<0.05) in comparison to negative controls. The experiments are representative of mean +/- SEM of two different experiments done in triplicates.

In order to study the production of cytokines, different concentrations of vaccine construct were used to elicit RAW264.7 and THP-1 macrophages. Culture supernatant was collected from unstimulated as well as *Ld*MAPV, LPS, and *Ld*6PGDH treated cells, succeeded by estimation of various cytokines through sandwich ELISA. The RAW264.7 cells treated with purified *Ld*MAPV resulted in the production of 0.5 to 1.5 ng of TNF-α (**Figures 9A, B**), whereas 0.5–0.8 ng of IL-1β, and 0.5–0.6 ng of IL-6 were found to be generated (**Figures 9C–F**). Notably, IL-10 (Th2 cytokine) was observed in the range of 0.2–0.6 ng against different concentration of the vaccine protein (**Figures 9G, H**). The levels of TNF-α were between 0.5 and 0.6 ng up to 2 µg/ml of vaccine protein stimulation and increased to 1.5 ng with 5 and 10 µg/ml, whereas IL-1β was found to be increased according to the concentration of *Ld*MAPV. Similarly, THP-1 macrophages also generated TNF-α, IL-6, IL-12 and IL-10 cytokines upon elicitation through *Ld*MAPV, with respective levels as 0.8–1.2, 0.5–0.8, 0.1–0.5, and 0.1–0.25 ng (**Figure 10**). The rising amount of IL-12 was observed to be dependent on the dose of *Ld*MAPV with heightened response by the cells stimulated for 48 hours, but TNF-α and IL-6 did not upsurge corresponding to the increased time or concentration of vaccine candidate. Moreover, generation of the anti-inflammatory cytokine, IL-10 was lower than that of the pro-inflammatory ones tested here. *Ld*6PGDH did not stimulate the synthesis of any cytokine in the cells, while LPS induced the production of all the tested ones. Hence, the enhanced cytokine levels in mammalian cells suggest an *in vitro* immune response by the designed vaccine.

## 4 Discussion

Vaccination is considered to be the most common approach to preventing infections caused by pathogens. The traditional methods for developing vaccines are quite costly and arduous, as well as posing a high risk of failure. Immunoinformatic-directed vaccine formulation might expedite the process by identifying prospective epitopes from proteins that can be utilized to construct vaccine candidates (40). As the multi-epitope vaccines are designed to enhance the summative effect of cellular, humoral, and innate immune responses, they possess an edge over the monovalent candidates/formulations (41). Recently, several attempts have been made to employ the immunoinformatics approach for designing and assessing multi-epitope vaccine constructs for leishmanial parasites (42–46). In this study, a new multi-epitope vaccine candidate has been proposed from *Ld*MAcP that plays a substantial role in parasitic virulence. It doesn’t show significant similarity with the human proteome, which suggest an extremely lower possibility of triggering the autoimmune response. Remarkably, the *Ld*MAcP sequence was found to be antigenic and non-allergenic with appropriate TM helices, indicating that this protein is a good vaccine candidate against VL. Since both types of immunity, i.e., T-lymphocyte effector response and long-lasting B-cell memory, are essential for protection from the parasite (47, 48), four CTL as well as HTL epitopes were screened based on immunogenic, antigenic, non-toxic, non-allergenic, and MHC-I and II binding allele characteristics. The activation of macrophages is necessary for eradicating the microorganism *in vivo* with IFN-γ as the main macrophage-activating factor (49). Notably, all selected HTL epitopes have presented the ability to induce IFN-γ. In addition, the B-cell epitopes of the designed vaccine candidate could elicit a humoral as well as a cell-mediated immune response. Nevertheless, previous studies on leishmanial multi-epitope vaccines were mainly focused on specific T-cell epitopes and didn’t consider B-cell epitopes for vaccine development (50, 51). Simultaneously, all the selected epitopes of the designed vaccine revealed no similarities with the human proteome, delineating its efficacy to stimulate vigorous immune reactions and excluding plausible deleterious allergic responses. The selected T-cell epitopes have shown coverage of more than 90% of Indian and Sudanese allelic populations, suggesting that the proposed vaccine could help to fight against VL in most of the affected countries of the world.

It is crucial to keep the synthetic protein small in size to reduce the production cost, enable its purification from inclusion bodies, and avoid toxicity in the host organism (52). Hence, only ten epitopes were chosen for designing a vaccine where overlapping epitopes were not included to maximize the total number of epitopes in the construct without increasing the length. Moreover, the proposed vaccine delineated a better immune response than the previously reported multi-epitope constructs, which were longer but harbored fewer epitopes in their sequence (42, 43). The screened epitopes were joined to obtain the vaccine construct through appropriate linker sequences that facilitate the formation of the natural structure of epitopes with enhanced folding and stability, leading to an ultimate upsurge in multi-epitope vaccine recognition. Moreover, RS-09 peptide (a TLR4 agonist) was incorporated as an adjuvant to the N-terminus of the designed sequence that binds to TLR4 and stimulates dendritic cells, resulting in the generation of tumor necrosis factor-alpha (TNF-α) and interleukin-1 beta (IL-1β) secretion (53). Further analysis revealed that the vaccine candidate is antigenic and non-allergenic, suggesting that the meticulously designed construct might be safe in *in vivo* assays. The residues of the epitopic region elucidate distinct secondary structural elements possessing α-helices predominantly. A three-dimensional structure of vaccine protein was generated and that presented better quality than the ones obtained from previously designed multi-epitope leishmanial vaccines (45, 54, 55). As the conformational stability of folded proteins depends upon several interactions, including disulfide bonds (56), disulfide-engineered vaccine protein showed more physiochemical and structural stability that was further evidenced by molecular dynamics simulation. Additionally, the final vaccine construct comprises both linear and discontinuous epitopes that can imitate the condition of actual infection, which in turn induces the production of antibodies.

Involvement of TLRs, primarily TLR2 and 4, is known to elicit differentiation of T helper (Th-1) cells that are engaged in significant mitigation of leishmaniasis (57, 58). The interacting patterns and docking scores implicated the strong affinity and structural stability of the designed vaccine towards TLR2 and TLR4 receptors. It was also observed that both TLR4 and MD2 were accountable for stable interaction with *Ld*MAPV, which is in accordance with the earlier study (11). Moreover, molecular dynamics simulation studies revealed that vaccine in complex forms possesses a sturdy profile with very few fluctuations, defining the stable nature of the vaccine in complex forms. Further analysis of hydrogen bonds concluded that the *Ld*MAPV-TLR4 complex had a higher number of hydrogen bonds as compared to the *Ld*MAPV-TLR2 complex, which was also comparable with the previous report (59). *In silico* immune simulation is a well-known method to establish the immune response through specialized cells mimicking infection conditions. Moreover, the immunological profile of the vaccine protein was also compared with the leishmanial A2 protein that was developed as Leish-Tec^®^ vaccine, the only vaccine commercially available for canine visceral leishmaniasis in Brazil (60). The proposed vaccine showed a strong cellular and antibody-based immune response in comparison to the A2 protein.

Codon optimization is critical for higher expression as gene expression varies across different hosts due to the inconsistency of the mRNA codons (61). Appropriate CAI value and GC content of the codon-optimized vaccine protein advocate higher expression in the bacterial system. The successful purification and subsequent western blot analysis of recombinant protein further validated the computational predictions of codon usage, overexpression, and solubility of the designed vaccine. Similarly, immunoinformatics studies also indicated the non-toxicity of *Ld*MAPV, which was further supported by our *in vitro* experiment demonstrating no adverse effects on the proliferation of mammalian macrophages. Host immune responses are primarily regulated by various immune cell types that secrete multiple cytokines and chemokines, which interconnect inflammatory and immune responses (Th1 and Th2) against microorganisms that cause infections (62). Here, *Ld*MAPV was found to significantly increase TNF-α, IL-1β, IL-6, and IL-12 cytokines that was in accordance with previous studies where *in silico* designed multi-epitope vaccines for VL were effective in inducing immunogenicity by upregulating the pro-inflammatory cytokines such as IFN-γ and TNF-α (63, 64). Similarly, in accordance to the previous report (64), lower level of IL-10 (anti-inflammatory cytokine) was observed suggesting potential induction of Th1 type immune response through designed vaccine. Nitric oxide (NO) is a critical effector molecule derived from immune cells that functions as a toxic agent against many noxious infections, including leishmaniasis, trypanosomiasis, malaria, and toxoplasmosis (65). Notably, mouse macrophages stimulated with *Ld*MAPV exhibited a significant increase in the production of intracellular NO that is in agreement with a vaccine study on mice immunized with a cocktail of epitopes targeting leishmaniasis (66).

## 5 Conclusion

Leishmaniasis is not only a health problem but also a disease related to social and economic conditions; hence, the development of a potent and efficient vaccine could be a way forward to successfully combat this disease. This computer-aided study aimed to design a multi-epitope vaccine for leishmaniasis by accurately predicting immunodominant epitopes from the membrane-bound acid phosphatase of *Leishmania donovani*. The developed vaccine construct demonstrates acceptable antigenic, allergenic, physicochemical, and structural properties, whereas its interaction analysis with human toll-like receptors also confirms the stability of the complex. The immunological profile suggests that this vaccine is immunogenic and has the potential to induce strong T- and B-cell immune responses. Subsequently, the vaccine protein was purified to homogeneity and didn’t demonstrate a cytotoxic effect on macrophages. *In vitro* studies with purified vaccine established its role in eliciting an immune response through various cytokines, which needs further validation in animal models to evaluate its potency against leishmanial parasites.

## Data availability statement

The original contributions presented in the study are included in the article/**Supplementary Material**. Further inquiries can be directed to the corresponding author.

## Ethics statement

Only human/animal cell lines were used in the study after receiving approval of Institutional Biosafety Committee (IBSC), University of Hyderabad, Hyderabad, India with Ref. No. IAQN-1-August 2020.

## Author contributions

J: Data curation, Formal analysis, Investigation, Methodology, Software, Writing – original draft. RQ: Formal analysis, Investigation, Methodology, Writing – original draft. IQ: Conceptualization, Funding acquisition, Supervision, Writing – review & editing.
