# Supplementary material for: Development of a multi-epitope vaccine candidate for leishmanial parasites applying immunoinformatics and in vitro approaches
Source: Front Immunol. 2023 Nov 15;14:1269774. doi: 10.3389/fimmu.2023.1269774 (PMC10684680; doi:10.3389/fimmu.2023.1269774)
Supplement: Supplementary file 1 [file DataSheet_1.docx]

Supplementary Material

**Development of a multi-epitope vaccine candidate for leishmanial parasites applying immunoinformatics and *in vitro* approaches**

Jyotisha, Rahila Qureshi and Insaf Ahmed Qureshi*****

^a^Department of Biotechnology & Bioinformatics, School of Life Sciences, University of Hyderabad, Prof. C.R. Rao Road, Hyderabad 500046, India

^b^Centre for DNA Fingerprinting and Diagnostics, Hyderabad 500039, India

*****To whom correspondence should be addressed:

Dr. Insaf Ahmed Qureshi

Department of Biotechnology & Bioinformatics

School of Life Sciences, University of Hyderabad

Hyderabad 500046, India

E-mail: [insaf@uohyd.ac.in](mailto:insaf@uohyd.ac.in)

Phone: +91-40-23134588

ORCID ID: 0000-0001-7720-7067

**Supplementary Tables**

**Table S1: Comparative analysis of vaccine and disulfide engineered construct**

| **Vaccine construct** | **Antigenicity**  **(Vaxijen)** | **Allergenicity (AllerTop)** | **Solubility** | |
| --- | --- | --- | --- | --- |
|  |  |  | **(SOLpro)** | **(Protein-Sol)** |
| Apo vaccine | 0.9551 | Non-allergen | 0.954124 | 0.580 |
| Disulfide mutated vaccine | 0.9658 | Non-allergen | 0.959321 | 0.591 |

**Table S2: RMSD, Rg, and RMSF values for apo *Ld*MAPV and *Ld*MAPV-TLRs complexes**

| **Parameters** | **Apo *Ld*MAPV** | | | ***Ld*MAPV-TLR2** | | | ***Ld*MAPV-TLR4/MD2** | | |
| --- | --- | --- | --- | --- | --- | --- | --- | --- | --- |
|  | **Min (nm)** | **Avg (nm)** | **Max (nm)** | **Min (nm)** | **Avg (nm)** | **Max (nm)** | **Min (nm)** | **Avg (nm)** | **Max (nm)** |
| RMSD | 0.11 | 0.842 | 1.03 | 0.12 | 0.78 | 1.03 | 0.10 | 0.62 | 0.78 |
| Rg | 1.56 | 1.874 | 2.09 | 1.55 | 1.88 | 2.05 | 1.56 | 1.81 | 1.91 |
| RMSF | 0.19 | 0.42 | 0.86 | 0.13 | 0.36 | 1.05 | 0.10 | 0.26 | 1.17 |

**Supplementary Figures**

**
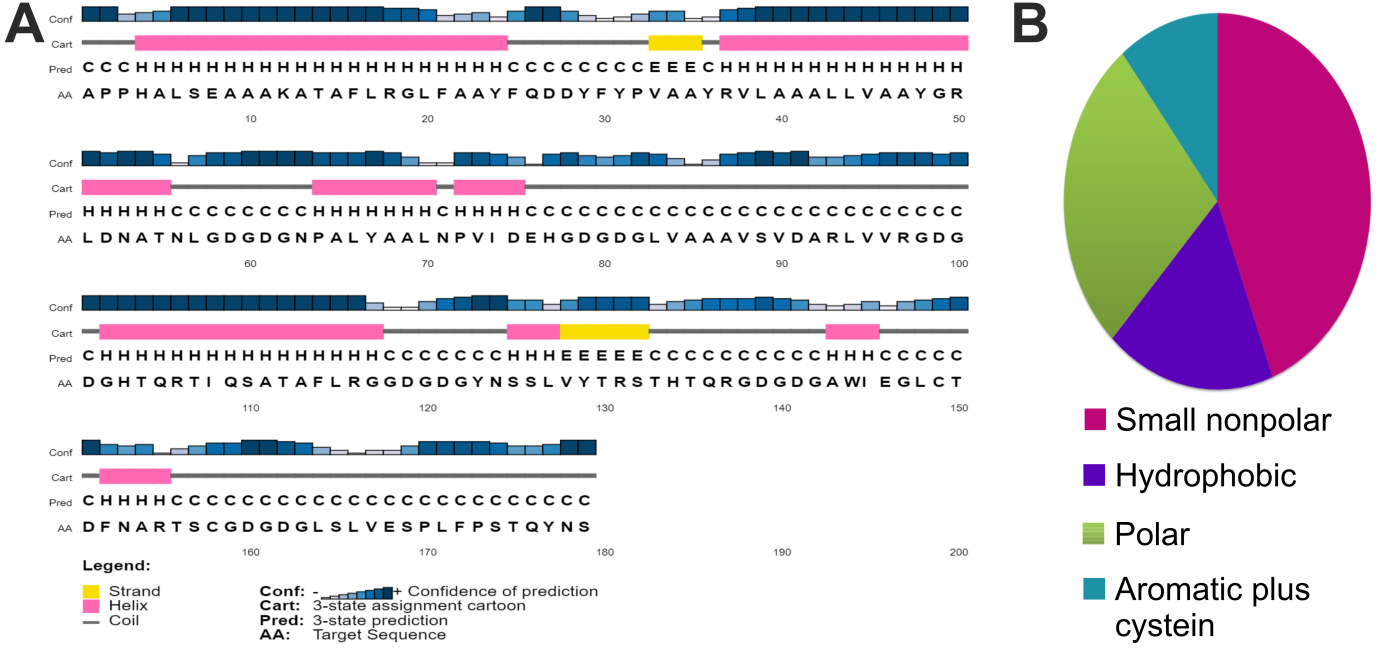
**

**Figure S1:** (A) Secondary structure analysis of *Ld*MAPV and (B) Nature of residues by PsiPred server.

**
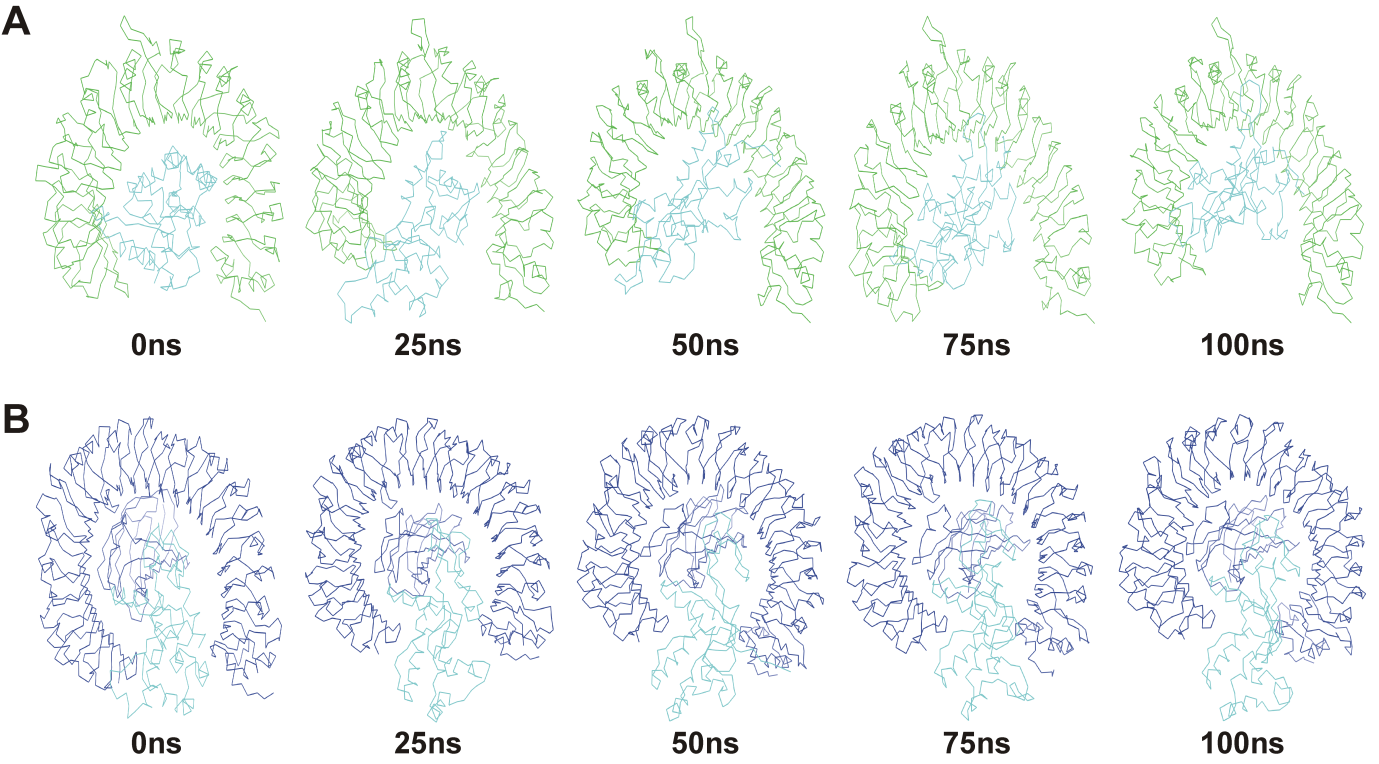
**

**Figure S2:** Changes in the structures of *Ld*MAPV-TLR2 (A) and *Ld*MAPV-TLR4/MD2 (B) at various time periods during molecular dynamic simulation.

**
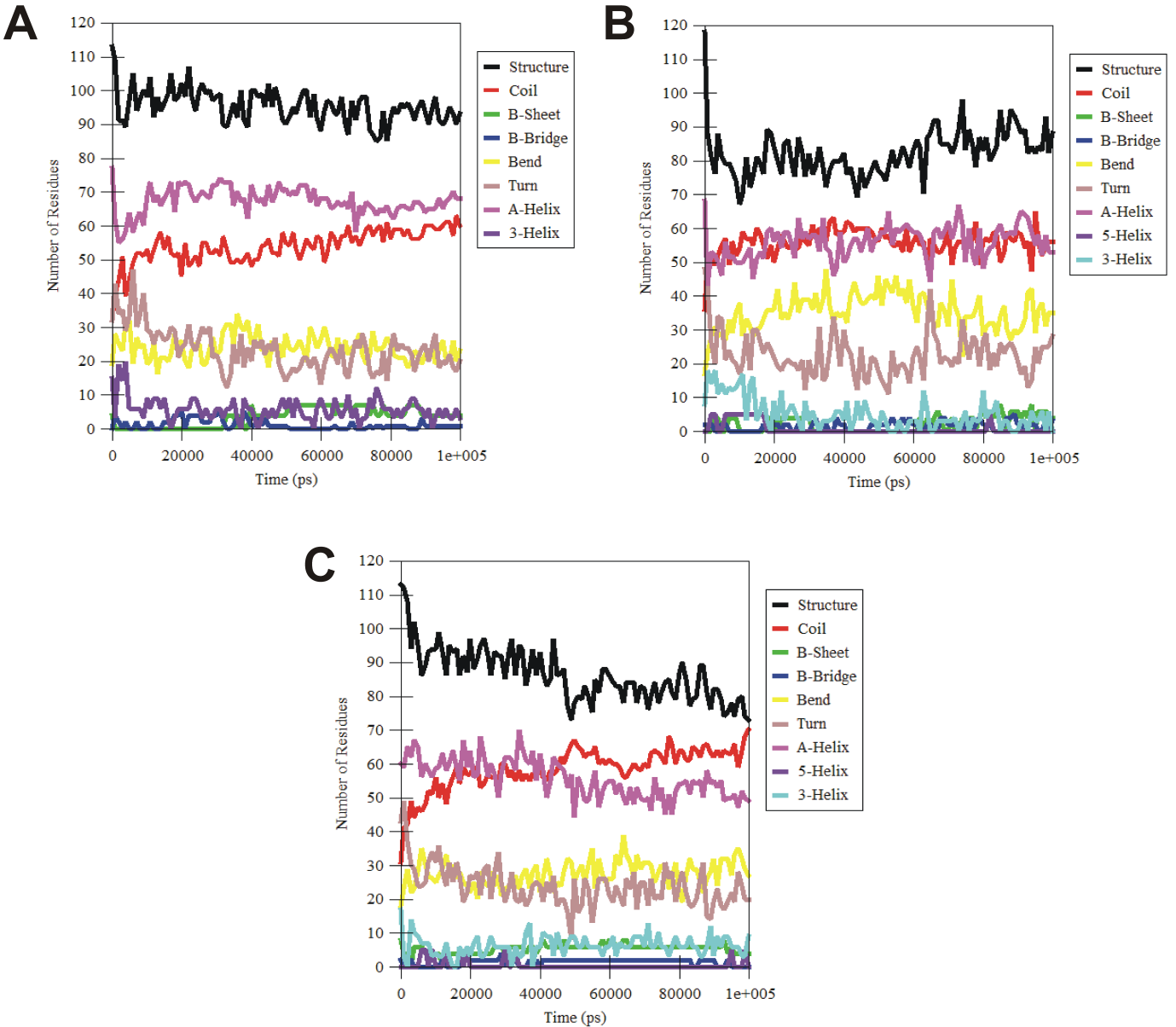
**

**Figure S3:** Secondary structure content assessment of apo *Ld*MAPV (A) and its complexes with TLR2 (B) and TLR4/MD2 (C) during dynamic studies of 100 ns.

**
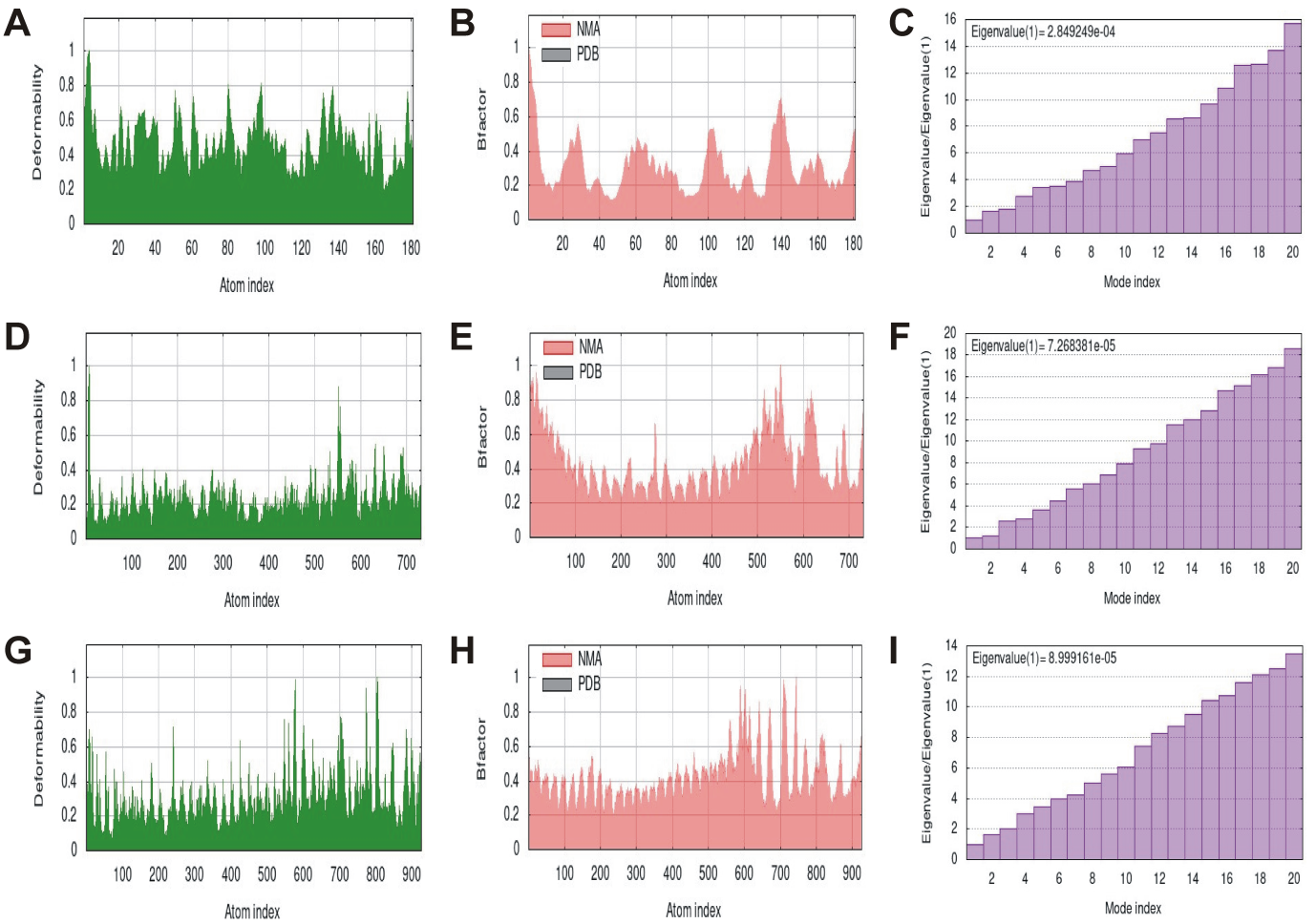
**

**Figure S4: iMODS server evaluation.** Deformability plot of atomic fluctuation depicting green-colored hinges for apo *Ld*MAPV (A), *Ld*MAPV-TLR2 (D) and *Ld*MAPV-TLR4/MD2 (G). B-factor graph of apo *Ld*MAPV (B) and its complexes with TLR2 (E) and TLR4/MD2 (H). The eigenvalue analysis for *Ld*MAPV in apo form (C) and with complex of TLR2 (F) and TLR4/MD2 (I)

**
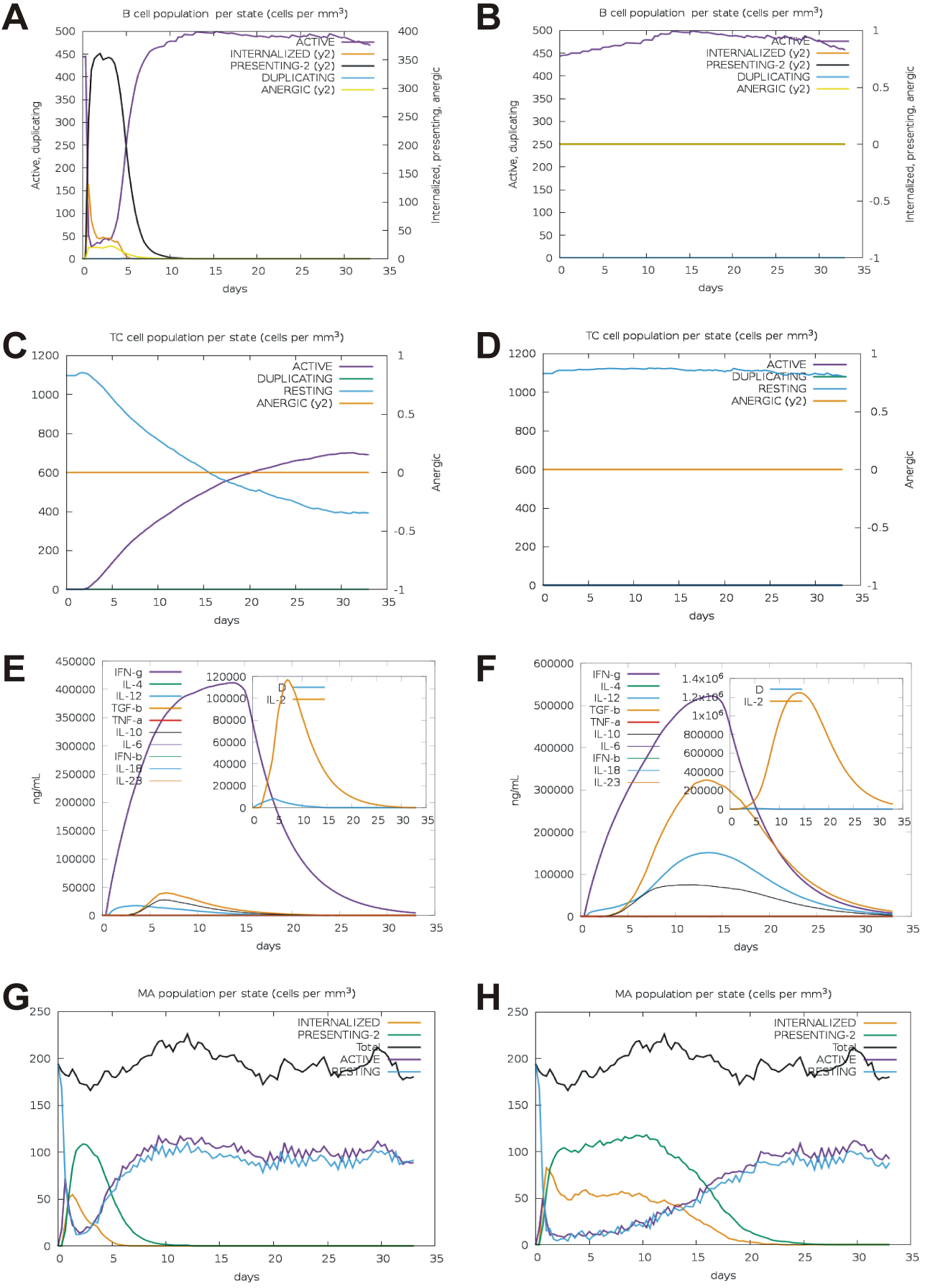
**

**Figure S5:** Prediction of immune response against the designed chimeric construct and reference protein. Active B-cell population for vaccine candidate (A) and A2 protein (B). Active TC cell population for designed vaccine (C) and A2 protein (D). The level of cytokines for vaccine candidate (E) and A2 protein (F). Macrophage cell population for designed vaccine (G) and A2 protein (H).

**
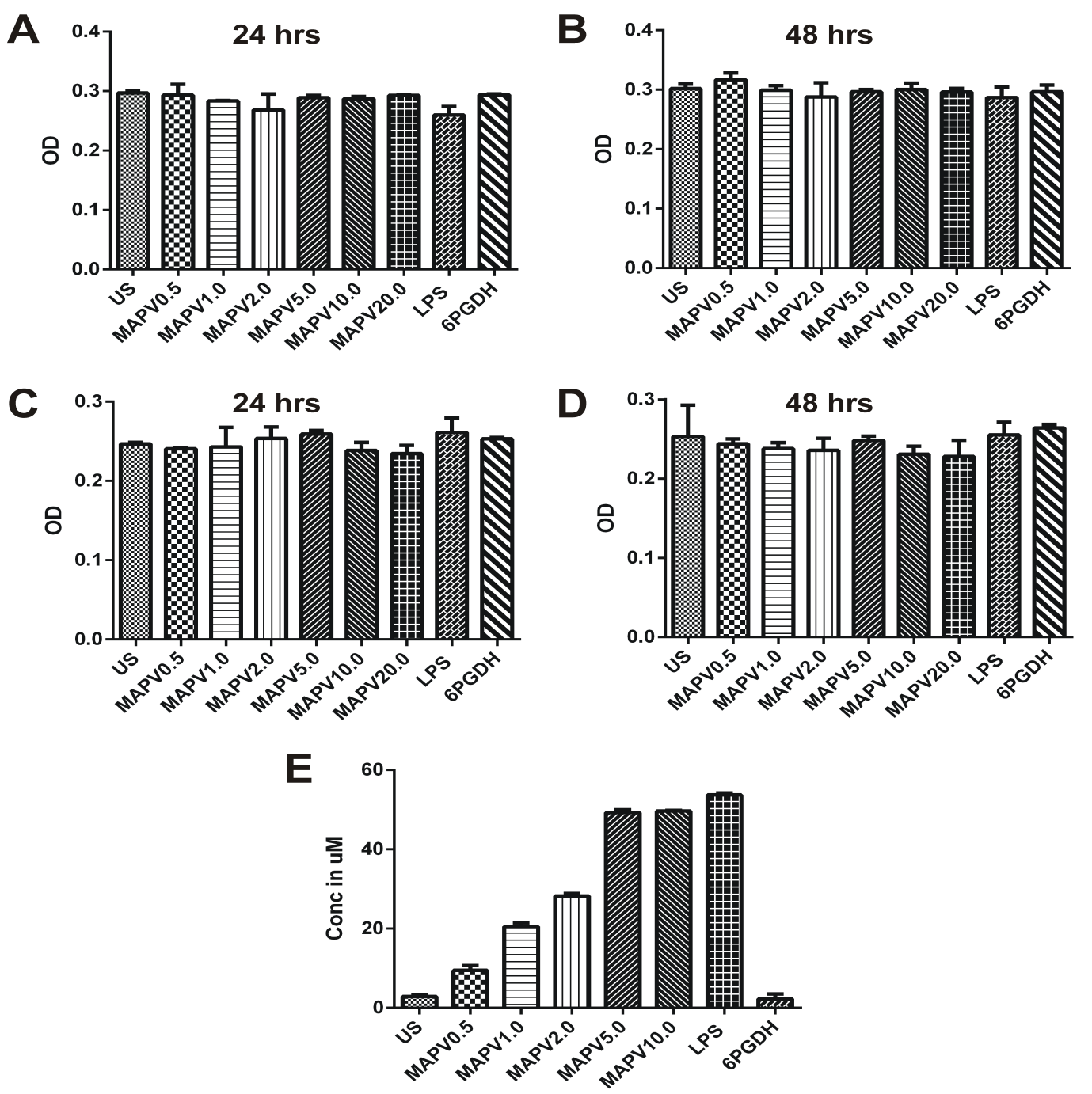
**

**Figure S6:** **Cell viability and nitric oxide generation by *Ld*MAPV.** RAW 264.7 (A-B) and THP-1 (C-D) macrophages were treated with purified *Ld*MAPV (0.5, 1, 2, 5, 10 and 20 µg/ml), *Ld*6PGDH (10 µg/ml) or LPS (1 µg/ml) individually and then MTT assay was performed after 24 and 48 hrs. Cells without any stimulation served as reference. (E) Murine macrophages RAW 264.7 were treated with *Ld*MAPV succeeded by NO evaluation in culture supernatant after 24 hrs using Griess reagent. Cells without any stimulation and treated with *Ld*6PGDH served as negative control, while LPS stimulated cells were taken as positive control. Difference in NO generation between stimulated and unstimulated cells is significantly evident with p values lesser than 0.05. The experiments are representative of mean +/- SEM of two different experiments carried out in triplicates.
